# *Boschniakia rossica* polysaccharides: extraction, structural features, experimental bioactivities, evidence limitations, and application prospects

**DOI:** 10.3389/fphar.2026.1905176

**Published:** 2026-09-03

**Authors:** Shuo Wang, Xiangming Sun, Fang Tao, Xuejing Yang, Lei Yu, Lang Lang, Jiahui Ma, Hui Song

**Affiliations:** 1 Center of Pharmaceutical Engineering and Technology, Harbin University of Commerce, Harbin, Heilongjiang, China; 2 The Second Hospital of Heilongjiang Province, Harbin, Heilongjiang, China

**Keywords:** *Boschniakia rossic*a, evidence limitations, experimental bioactivities, extraction, polysaccharides, structural features

## Abstract

*Boschniakia rossica* (Cham. and Schltdl.) B. Fedtsch. (BR) is a traditional medicinal plant that grows primarily in northeastern China. It is recognized as a representative traditional medicinal plant and has long been used in folk medicine for tonifying the kidney essence, relieving constipation, and strengthening the body. *Boschniakia rossica* polysaccharides (BRPs) are important bioactive components exhibiting multiple pharmacological activities. Existing studies indicate that BRPs are mainly obtained *via* solvent heat reflux extraction, ultrasonic-assisted, and ultrasonic-enzyme-assisted extraction. Structural features of the polysaccharide, such as the molecular weight, monosaccharide composition, and glycosidic bond linkages, are markedly affected by different extraction and purification parameters. Reported experimental bioactivities of BRPs include antioxidant activity, immune regulation, tumor-cell growth-inhibitory and pro-apoptotic effects, inflammation modulation, and liver protection. Building on a review of previous work, this study takes a closer look at how the preparation methods, structural traits, reported biological effects, and application constraints are connected. That said, the studies currently available cover a mix of experimental types—chemical assays, cell-based tests, and animal trials—which differ markedly in both the strength of their evidence and their value for pharmacological interpretation. Moreover, the exact structure–activity relationships, direct molecular targets, receptor-mediated mechanisms, long-term safety, and clinical applicability of BRPs have yet to be firmly established. In the future, more research efforts will be directed at the standardization of preparation protocols, deep structural characterization, mechanism-driven functional exploration, and translational applications of these polysaccharides in natural drugs and functional foods.

## 1 Introduction

**FIGURE 1:**
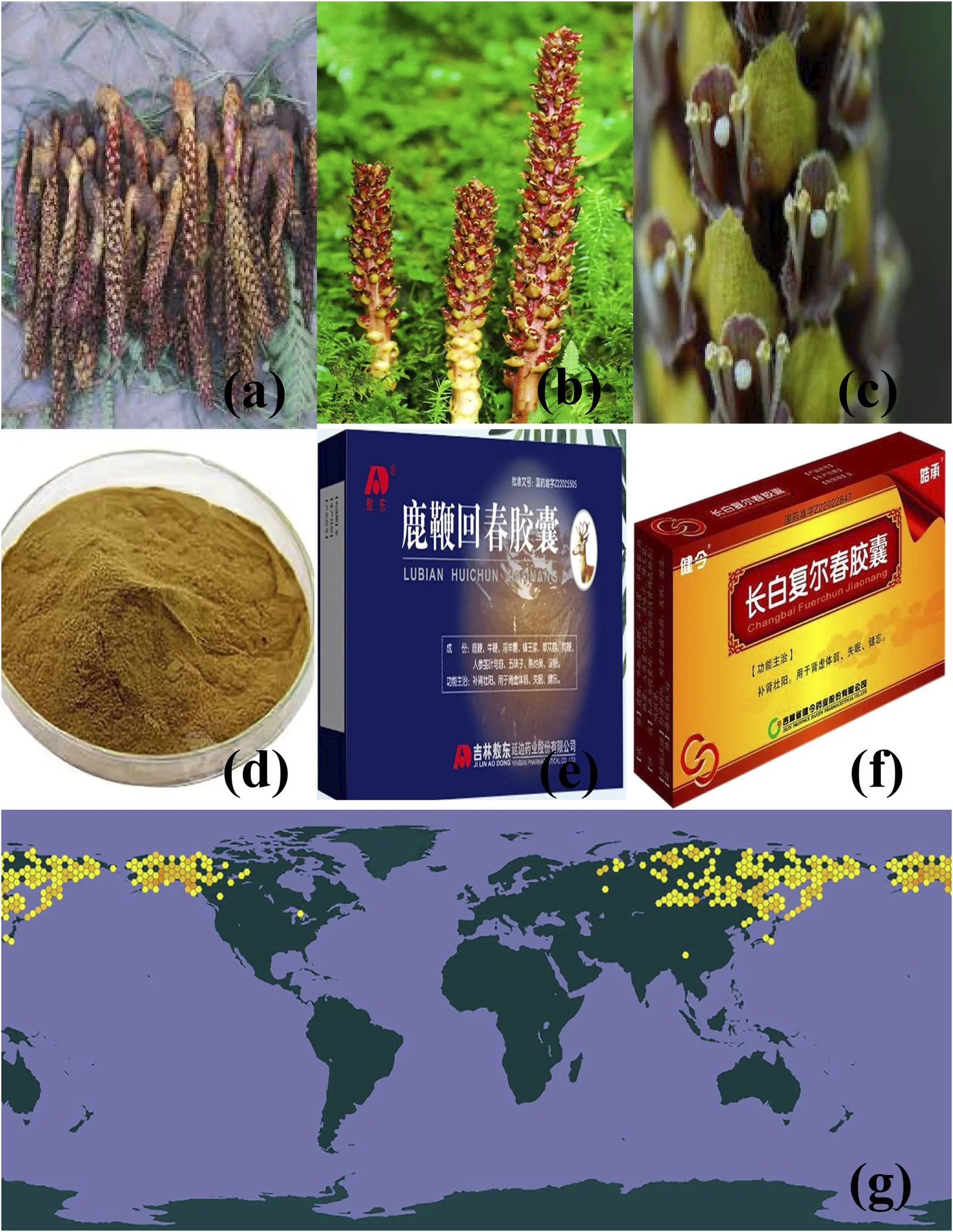
Distribution, botanical characteristics, and medicinal applications of *Boschniakia rossica*. **(a)** Whole plant. **(b)** Aerial parts. **(c)** Inflorescence. **(d)** Powdered crude drug. **(e,f)** Representative traditional Chinese patent medicines. **(g)** Global geographical distribution map.

*Boschniakia rossica* (Cham. & Schltdl.) B. Fedtsch. (BR) is a plant species and not a ‘botanical drug’ (Hu et al., 2025) belonging to the genus *Boschniakia* of the family Orobanchaceae (Ma et al., 2015). Historically revered as the “immortality herb” in folk medicine (Yi et al., 1999), it typically parasitizes the fine roots of *Alnus* species (Betulaceae), particularly *Alnus mandshurica* (Callier) Hand.-Mazz. [Betulaceae] (Zheng et al., 2010). BR is distributed mainly in the northern mountainous regions of the Greater Khingan Range (including Heilongjiang and Inner Mongolia) (Chen et al., 2015), along with the alpine birch forests and upper coniferous forest zones at altitudes of 1,350 m–2,000 m in the geothermal valleys of the Changbai Mountain area in Northeast China (Yu et al., 1996). At similar latitudes outside China, natural distributions of BR have also been reported in Siberia (Russia), the northern mountainous regions of Korea, and the Mount Fuji region in Japan (Figure 1g). Ethnopharmacologically, BR has been commonly used for the treatment of senile habitual constipation, kidney deficiency-induced impotence, cystitis, and infertility, and it also exhibits cardiotonic effects (Li C. F. et al., 2014; Wang et al., 2018). Recent experimental studies have reported several biological effects of BR, including anti-aging-related (Wang and Song, 2009), anti-inflammatory (Ji et al., 2010), and tumor-related activities (Shyr et al., 2006). Historically, these bioactivities were primarily linked to its small-molecule constituents, such as phenylpropanoid glycosides (Jin A. H. et al., 2011) alongside iridoid glycosides (Xu et al., 2025), among which, *B. rossica* polysaccharides (BRPs) have recently emerged as a particularly crucial class of macromolecules responsible for its therapeutic effects (Song et al., 2025).

Polysaccharides are essential natural macromolecular polymers; although they have long served as structural supports and energy sources for life, scientists have increasingly recognized their roles in various other areas in recent years (Huang and Huang, 2020; Chang, et al., 1998). Polysaccharides derived from ethnic medicinal herbs are complex polymers with significantly varying molecular weights, formed by glycosidic bonds linking various monosaccharides. They play a pivotal role in regulating biological processes (Gao et al., 2025). The overall physicochemical behavior and bioactivities of plant polysaccharides are collectively governed by their structural characteristics, encompassing monosaccharide profiles, molecular weight distribution, glycosidic stereoconfigurations, backbone chain conformation, branching extent, three-dimensional spatial folding, and chemical modification patterns (Sun et al., 2023). Such structural traits jointly regulate the aqueous solubility, storage stability, and functional bioactivity of these carbohydrate polymers (Pan et al., 2025). Due to low toxicity and numerous documented biological activities, including (Chen H. et al., 2023) antioxidant, (Ic et al., 2015) tumor-cell growth-inhibition, (Yu et al., 2018) and anti-inflammatory effects, botanical polysaccharides have been applied to develop functional foods and are also being studied in medicinal research.

With the promulgation of the Local Food Safety Standard—*B. rossica* (DBS 23/016–2022), *B. rossica* has been authorized for application as a food raw material within Heilongjiang Province. BRPs have become the subject of attention for recent phytochemistry and pharmacology studies (Huang et al., 2021). In recent years, all-encompassing research has been conducted on the extraction and isolation, chemical structure, and pharmacological effects of BRPs. Hot-water extraction and alkaline extraction have been used in several ways to separate and purify the different polysaccharide components, including BRT, BWI, BWII, BJI, and BJII, from the inflorescences and roots of BR (Chang et al., 1997). Notably, these isolated fractions have different molecular weights and monosaccharide compositions; the extraction methods for these fractions are also different. Therefore, the structurally diverse BRP fractions have exhibited all kinds of target biological activities, including immunomodulatory, tumor-cell growth-inhibitory, and anti-aging-related effects, which provide preliminary clues for further functional evaluation and product development.

However, most previous studies on BRPs have focused on optimizing the extraction process, characterizing individual structures, or verifying specific pharmacological activities. Systematic summaries of the extraction and isolation methods, structural features, pharmacological activities, and application developments of these are still absent. The relationship among the structure and activity of BRPs has not been fully revealed, and, therefore, their further research and application have been limited. This study does not only sort BRPs studies by publication date. After summarizing published data, we analyze the connections between preparation processes, structural traits, recorded bioactivities, and practical application barriers. We sum up how different extraction and purification approaches change BRP structures and combine the pharmacological results to discuss possible links between their structures and bioactive functions. We also address the unsolved problems in current research: incomplete structural identification, unclear structure–activity relationships, insufficient proof for direct molecular targets and receptor pathways, and scarce data on long-term safety and clinical effects. All these discussions can offer targeted ideas for standard BRP production, mechanism exploration, safety testing, and follow-up product research.

## 2 Methods

**FIGURE 2:**
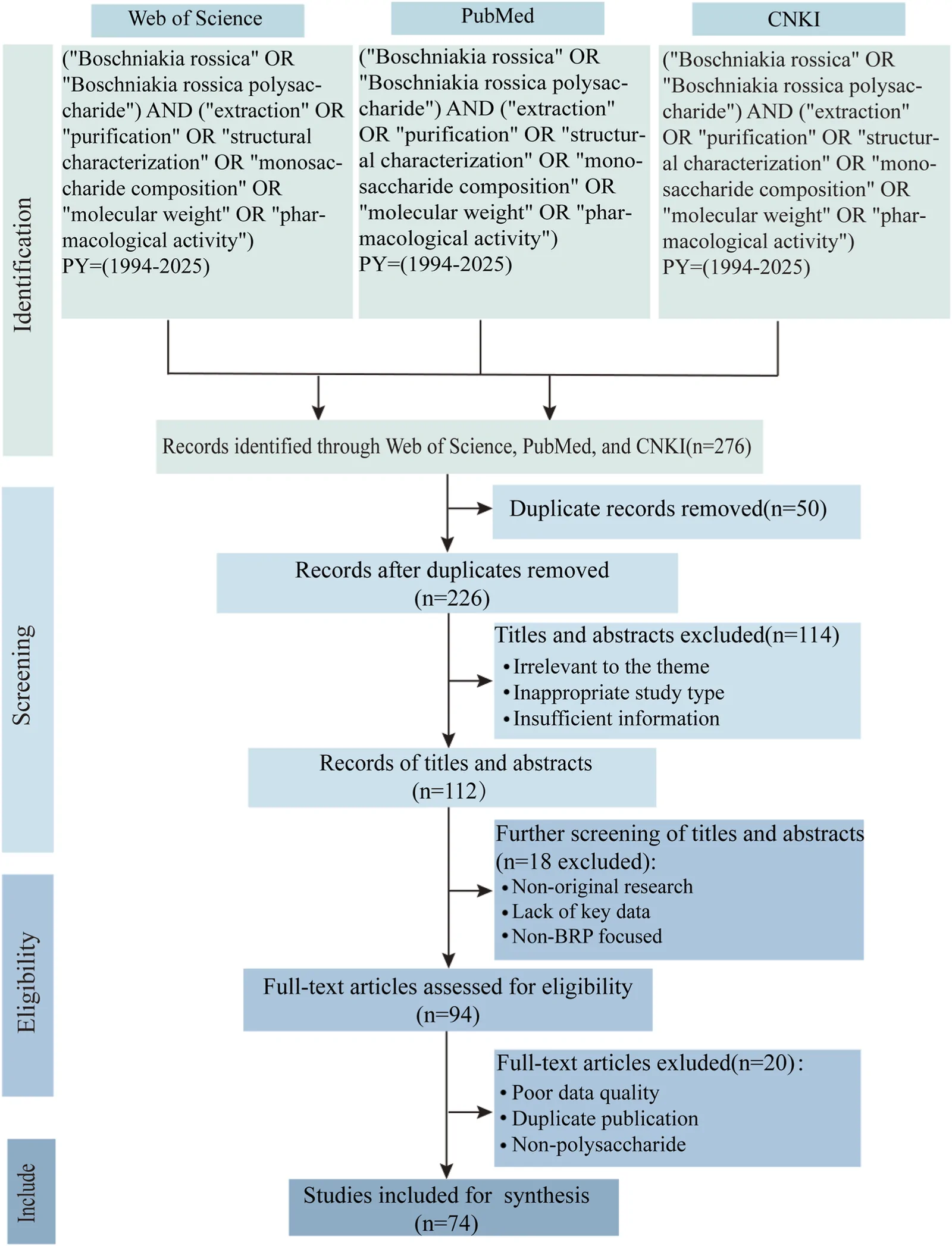
PRISMA flow diagram of the literature search and study selection process.

The preparation and reporting of this systematic review have been in line with the Preferred Reporting Items for Systematic Reviews and Meta-Analyses (PRISMA). To ensure the openness and reproducibility of the research, the same system was used for the literature search, screening, and quality assessment. Data were obtained from the Web of Science, PubMed, and CNKI databases. Search strategies combined the following search terms: (“*Boschniakia rossica*” OR “*Boschniakia rossica* polysaccharide”) AND (“extraction” OR “purification” OR “structural characterization” OR “monosaccharide composition” OR “molecular weight” OR “pharmacological activity”). A total of 276 potentially relevant literature records were obtained at the start of this study. After excluding duplicate documents and screening publications in accordance with the predetermined inclusion and exclusion criteria, a total of 74 research reports were finally included in this systematic review. All of the included literature were divided into four categories of research directions: ethnobotany, phytochemistry, pharmacology, and applications. In light of the above evidence, we have also considered the themes of relevance and research design, among others, in our analysis. A particular division was made to determine whether the research results were obtained from *in vitro* experiments, animal experiments, or human studies. The main assessment indicators are the appropriateness of the research plan, the size of the sample, the administration schedule for drugs, and how well the obtained results clarify the reasons in the body of disease. According to the official taxonomic materials, *B. rossica* has been listed in the databases for classification and distribution by reliable biodiversity databases. Therefore, this study will provide an organized and critical overview of the research evidence that has been collected, but it will not be a formal systematic review with meta-analysis. The entire process of literature collection, initial filtering, and final study selection are shown in Figure 2.

## 3 Ethnopharmacology of BR

### 3.1 Conventional applications

Across the traditional medical systems of the northern ethnic groups in China, such as Han, Mongolian, and Oroqen medicine, BR is frequently used to replace *Cistanche deserticola* (CD) species. The first two functions of this are to strengthen the kidneys and treat sexual dysfunction. In addition, it has been used to treat neurasthenia, palpitations, insomnia, senile chronic constipation, and deficiency-related cough, and it is reported to have benefits for bone hyperplasia and female infertility. Early forms of administration included decoctions, medicinal wines, medicated diets (stewed preparations), and topical applications (Zhang H. et al., 2016).

BR, also known as Bullocho in Korea, is a whole-plant medicinal material in traditional Korean medicine that generally has properties of kidney deficiency. It is also used to treat erectile dysfunction, spermatorrhea, cold pain in the lower back and knees, metrorrhagia, and cystitis. In Mongolian medicine, it is known as “Bao Ri-Gao Yao,” and the whole botanical drug is used to treat acid regurgitation, abdominal distension, headache, leukorrhea, and lumbago. In short, the consensus of the above-mentioned diverse ethnopharmacological systems is that it can strengthen the kidney-yang, relieve constipation, and exert a hemostatic and analgesic effect (Bai et al., 2014). The specific ethnic medicinal applications, traditional names, and preparations of BR in various medical systems are listed in Table 1.

**TABLE 1:** Traditional and ethnopharmacological uses of *Boschniakia rossica*.

Traditional medical system	Traditional name	Part used	Traditional actions	Indications	Preparation and administration
Korean medicine	Bullocho (Bu Lao Cao)	Whole Plant	Nourishing kidney-yin	Kidney-yin deficiency, impotence, spermatorrhea, lumbago due to cold pain, constipation, uterine hemorrhage, and cystitis	Decoction (oral administration)
Mongolian medicine	Borjigao (Bao Ri-gao Yao)	Whole Plant	Tonifying kidney-yang, relieving constipation, arresting bleeding, and relieving pain	Acid regurgitation, abdominal distension, headache, impotence, abnormal leukorrhea, and lumbago	Decoction (oral administration)
Han medicine	Cao cong rong	Whole Plant	Tonifying kidney-yang, moistening the intestines and relaxing the bowels, and tonifying deficiency	Impotence, neurasthenia, palpitation, insomnia, senile chronic constipation, deficiency cough, osteoproliferation, and female infertility	Decoctions, medicinal wines, stewed preparations (medicated diets), and topical applications

### 3.2 Medicinal historical context

The historical development of “Caocongrong” can be traced back to classical Chinese pharmaceutical texts. The earliest known textual appearance of it dates back to the Tang Dynasty and is included in *the Newly Revised Materia Medica* (新修本草). The above classic mentioned this herb in the annotation of the Commentaries on *the Divine Husbandman’s Classic of Materia Medica* (神农本草经集注) under the entry for CD, describing it as “short in shape with sparse flowers.” It also noted that ancient physicians frequently removed its flowers and used them as a substitute for CD, although their therapeutic effects were significantly weaker than those of the genuine product (Niu et al., 2023; Li J. W. et al., 2014). In the Song Dynasty, *Kaibao Materia Medica* (开宝本草) recorded that “Orobanche grows on rocks south of the mountains and looks like lotus root; it is also called Lielidang or Caocongrong.” *The Illustrated Classic of Materia Medica* (本草图经) further confirmed that “the root of Caocongrong is very close to that of Roucongrong; after deflowering and pressing, it can be used as a fake with significantly reduced effectiveness, and it is actually Orobanche” (Tu et al., 1994). In the Qing Dynasty, *Yijing Yunzhong* (医镜云踪) also said that “Caocongrong is also known as Orobanche.”

At present, the contents of these documents have begun to change from pure identification to practical medical application. At approximately 1961, in *Wild Economic Plants of Jilin Province* (吉林省野生经济植物), BR was first recorded as a medicinal plant, and it was found that its whole herb could be used to treat spermatorrhea, impotence, cystitis, and bleeding of the bladder and kidney; it was also applied as a veterinary tonic for invigorating essence. Later works, such as *Jilin Chinese Medicinal Herbs* (吉林中草药) in the 1970s, have also mentioned that it has been used to treat impotence and chills in the loins and knees caused by renal insufficiency, along with dryness of the bowel leading to constipation. By the early 1980s, *Materia Medica of Changbai Mountain Plants* (长白山植物药志) had added the clinical indications for female infertility, metrorrhagia, leukorrhagia, and strangury with residual urine, thus building an all-round medicinal application system. Later developments focused on both regional applications and scientific applications. In the late 20th century, works such as *Medicinal Plants of Northeast China* (东北药用植物) and *Inner Mongolia Materia Medica* (内蒙古植物药志) added to the study of cardiotonic properties and recorded their application in Mongolian medicine, providing a standardized system for dose and administration across ethnic groups. In the 1990s, extensive compilations were published, such as *the National Compilation of Chinese Herbal Medicines* (全国中草药汇编) and *Chinese Materia Medica* (中华本草), which systematically collected the plant origins, properties, and effects of botanical drugs and formally clarified the difference between the ancient and modern concepts referred to as “Caocongrong.”

In the 21st century, regional floras, such as the *Flora of Heilongjiang Province* (黑龙江省植物志) have described their ecological features and parasitic habits; later works have added modern pharmacological evidence of antioxidative, immunomodulatory, anti-aging, and anti-tumor activities. By 2019, this progression led to the *Jilin Provincial Standard for Chinese Medicinal Materials* (吉林省中药材标准); BR was formally recognized as a widely used ethnic botanical drugs, its harvesting and processing methods were standardized, and its therapeutic functions were legally confirmed; thus, it could be subjected to standardized and regulated clinical trials.

### 3.3 Taxonomy

Accurate taxonomic identification is essential for distinguishing BR from closely related taxa and for ensuring consistency across ethnobotanical, phytochemical, and pharmacological studies. According to Plants of the World Online (POWO), BR belongs to the family Orobanchaceae and the genus Boschniakia.

## 4 Habitat and morphological characteristics of BR

### 4.1 Habitat

BR is generally found in the cool, moist montane forest ecosystems at the range of 1,350 m–2,000 m (Chu et al., 2009). These areas are relatively cool all year round, often rainy, have a short growing season, and experience a large range of temperatures between day and night. Generally, the floor of a forest has a relatively thick layer of moss (approximately 10 cm–15 cm) and is, therefore, humid and oxygen-rich, and thus, it is suitable for the life cycle and growth of parasites. The species is mainly found in acidic mountain meadow forest soils with a relatively high water table. It can often be found in relatively open mixed stands of spruce and alder, and where there is a thick carpet of moss in the understory. As an obligate parasitic plant, it usually attaches to the fine roots of *Alnus mandshurica* (Yu et al., 1996).

### 4.2 Morphological characteristics

BR has a single, upright stalk that is approximately 15 cm–35 cm high and has a diameter of 0.5 cm–1 cm. The stem surface is dark yellowish-brown to brown, with longitudinal ridges and grooves, and sparsely covered with short white hairs (Zhang and Ding, 2002) (Figure 1b). It has reduced to a scale-like or lanceolate structure, is alternately arranged and slightly wrinkled, 0.8 cm–1.5 cm long, and yellow–brown in color. A terminal spike inflorescence is usually 5 cm–10 cm long and located at the top of the stem. Flowers are loosely arranged in the lower part and are denser at the top. Individual flowers are reduced in size after drying, but they appear in a bilabiate shape when fresh, are approximately 1.5 cm–1.8 cm long, and have a color range of yellowish-brown to light purple. Elliptical brown capsules (approximately 1 cm in length) have been found in some samples at the bottom of the spike. The calyx is a cup-shaped five-lobed part with irregular teeth, and the corolla is bilabiate and has a swollen sac-like tube. The lower lip is relatively short, has three lobes, and has fine hairs on the edge (Li and Zheng, 1985) (Figure 1c). It is a brittle plant that breaks easily, has an off-white fracture surface, and has irregularly spaced brown rings in the center.

## 5 Extraction and isolation processes

Various kinds of structural modifications of the polysaccharides are caused by different methods of extraction and purification (Wang and Li, 2025). Generally speaking, the four links in the extraction and isolation process of BRPs are pretreatment of raw materials, polysaccharide extraction, preliminary separation, and further purification. Among the above methods, the selection of an extraction method can affect the yield and structural integrity of polysaccharides significantly, and a poorly executed separation and purification process will also lead to less pure, structurally heterogeneous polysaccharides (Tian et al., 2019; Zhu et al., 2022). Therefore, both processes need to be optimized based on the physicochemical properties of BRPs. Polysaccharides are a type of natural bioactive substances obtained from all kinds of medicinal plants, and they are mainly distributed in the cell walls and mucilaginous layers of these plants (Yu et al., 2025). To increase the extraction efficiency of BRPs, generally speaking, the dried BR material is ground into a fine powder, and then ethanol is added for defatting to remove lipids, natural pigments, and other low-mass impurities (Gou et al., 2016). Many strategies have been proposed for the extraction of BRPs. Now, in addition to the old way of solvent extraction, some hybrid strategies that add enzymes and ultrasound are also being developed. At the same time, methods that use acids or alkalis, along with microwave and ultra-high-pressure technology, are being introduced one by one (Pan et al., 2025). Among the above methods, enzyme-assisted extraction has the advantage of mild conditions and high extraction efficiency, but it is also complex to operate and requires enzyme inactivation and removal; thus, it is not suitable for large-scale preparation (Xu et al., 2023). Acid- and alkali-treatments often induce the scission of glycosidic bonds and thus degrade the structure of polysaccharides; at the same time, cavitation effects under ultrasound can also alter the structure of polysaccharides (Xue et al., 2024). Therefore, we need to adjust the extraction plan based on the features of the BRPs.

### 5.1 Solvent extraction method

Water extraction uses distilled water as the solvent and relies on the water solubility of polysaccharides for extraction. Liu et al. (2011) extracted the residue of BR after it was defatted with 95% ethanol using 10 volumes of distilled water at 100 °C under reflux for four cycles of 180 min each. Crude polysaccharides were obtained after subsequent concentration, ethanol precipitation, deproteinization, and dialysis. This method is widely regarded as a simple, environmentally benign, and cost-effective approach, but it has several disadvantages, such as high levels of water-soluble impurities, low extraction efficiency, and easy mold-growth in the extract (Wang et al., 2022). Using single-factor experiments and an L9(3^4^) orthogonal experimental design, Liu (2013) identified the ideal circumstances for hot-water reflux extraction of BRPs: a solid–liquid ratio of 1:10, refluxing with petroleum ether twice (1 h each), refluxing with 80% ethanol twice (1 h each), extracting the residue with distilled water under reflux three times (1.5 h each), and precipitating the extract with ethanol at a final concentration of 90%; the final extraction yield was 7.95%.

Alkaline extraction involves mainly 0.1 mol/L–0.5 mol/L NaOH solution as the extractant, which can increase the dissolution rate of polysaccharides. However, strongly alkaline conditions tend to cause the rupture of glycosidic bonds and the breakdown of polysaccharide frameworks. Sun and Chang (2007) adopted a stepwise extraction method that combines water extraction and 0.1 mol/L NaOH-assisted alkaline extraction to isolate BRPs. Triple aqueous extractions were performed at 100 °C, with each reflux session lasting 120 min to process the raw material, after which, 500 g of the water-extracted residue was soaked in 2 L of 0.1 mol/L NaOH solution five times. Gradient experiments verified that 0.1 mol/L–0.3 mol/L NaOH was the suitable concentration for extracting polysaccharides from the rhizomes of BR, among which 0.2 mol/L NaOH yielded the highest dissolution rate of bound polysaccharides. Although 0.5 mol/L NaOH could increase the dissolution amount, it led to a decrease in the Mw of polysaccharides, mainly because of the cleavage of glycosidic bonds caused by the strongly alkaline environment (Song, 2006). This finding shows that although alkaline extraction can improve the yield, it may sacrifice the structural integrity of polysaccharides. Therefore, subsequent studies need to find a balance between the yield and structural preservation.

### 5.2 Ultrasound-assisted extraction

Plant cell walls are quickly and completely disrupted by ultrasound-assisted extraction, which makes use of the cavitation and mechanical vibration effects of ultrasound (Wang et al., 2023), thus promoting polysaccharide dissolution. In contrast to conventional single-step extraction, this approach greatly improves polysaccharide output and shortens the extraction time. Similar strategies have also been successfully applied in other polysaccharide systems, such as *Tremella fuciformis* (Yang et al., 2025). As an efficient method for BRP extraction, ultrasound-assisted extraction has gained extensive attention in the field of natural product research, and it is often combined with water extraction and enzymatic extraction. Song et al. (2025) adopted ultrasound-mediated extraction coupled with ethanol-based fractionation. This process was executed at ambient temperature, maintaining a 1:100 solid-to-solvent ratio over a 120-min duration, which ultimately yielded favorable polysaccharide recovery and established a clear linear correlation.

The main limitation of this method is that excessive ultrasound power generates local high temperatures, which may disrupt the polysaccharides’ higher-order structure (Lao et al., 2017) and reduce their biological activity. Therefore, the ultrasound power and extraction time needs to be tightly regulated in practical applications to balance extraction efficiency and the polysaccharide’s structural integrity.

### 5.3 Ultrasound-assisted enzymatic extraction

Recently, Yang et al. (2025) applied ultrasound-assisted enzymatic extraction (UAEE) for BRP extraction. A multi-enzyme preparation of cellulase, pectinase, and papain was selected. First, the optimal enzyme formula was obtained through orthogonal screening, and then extraction conditions were optimized using univariate analysis and response surface methodology (RSM). The best efficiency was achieved with a material-to-solvent ratio of 1:25 (g/mL) in an 80% ethanol medium at 73 °C, and a 450 W ultrasonic device was used for 100 min. Under the above target parameters, the recovery rate of raw polysaccharide reached 13.67%, and it was 1.52-fold and 1.17-fold higher than that obtained by ultrasonic-assisted (UAE) and enzymatic (EAE) methods alone. This way integrates the cavitation and mechanical effects of ultrasound with the biocatalytic effect of enzymatic hydrolysis to break down the cell wall of BR efficiently, increase solvent permeability, and achieve fast and highly efficient release of polysaccharides. Moreover, mild extraction conditions can better preserve the biological activities of polysaccharides. Single enzymatic extraction is specific for disrupting plant cell walls. It appears to be porous because of the visible breakdown and peeling caused by enzymatic deterioration. However, its ability to disrupt rigid cell walls depends mainly on the efficiency of the enzymatic reaction (Puri et al., 2012). Therefore, it still has certain application limitations in the large-scale preparation of BRPs. In the future, it is necessary to develop more efficient and low-cost enzymatic hydrolysis systems, together with optimized enzyme inactivation and removal processes, to balance extraction efficiency and production cost.

### 5.4 Other extraction methods

With the benefits of reduced sample consumption, quick extraction times, and high extraction efficiency, online pressurized solvent extraction is a new and effective extraction technique. Rapid profiling of chemical constituents in BR can be effectively achieved *via* this approach. Its reliance on aqueous extraction epitomizes the “green analytical chemistry” (Li et al., 2020). Compared to ultrasound-aided methods, this technique yields more robust chromatographic signals, reflecting enhanced performance.

Pressurized solvent extraction is one of the methods for extracting natural polysaccharides; microwave-assisted and ultra-high-pressure extractions are other typical types of extraction methods now used. The former is a high-speed, environmentally friendly solvent (Mo, 2005), and the latter can be carried out at or near room temperature (Wang et al., 2024); both are relatively gentle to prevent damage to the structure of polysaccharides caused by heat. However, the applications of these methods to BRP extraction have not been widely carried out yet. Together, the above new methods offer good choices; however, they have not been widely applied in the extraction of BRPs.

To summarize, hot-water immersion, enzymatic digestion, and ultrasonic-aided processes are all viable pathways for obtaining BRPs. The main factors influencing the extraction yield of BRPs include the material-to-solvent proportion, duration of extraction, temperature during extraction, amount of enzyme used, and power of the ultrasound. Different extraction methods have their own strengths and weaknesses. Consequently, it is essential to choose the most suitable extraction technique combined with the specific characteristics of BRPs, and optimizing the extraction process is necessary to ultimately increase the yield of BRPs. We have compiled a summary of the extraction techniques for BRPs and displayed them in Table 2.

**TABLE 2:** Comparison of different extraction methods and parameters for *Boschniakia rossica* polysaccharides.

Extraction methods	Solid–liquid ratio (w/v)	Time (min)	Temperature (°C)	Power (W)	Yield (%)	References
Water extraction	1:10	180	100	—	—	Liu et al. (2011)
Water extraction	—	360	100	—	—	Sun and Chang (2007)
Water extraction	1:10	210	—	—	7.89	Liu (2013)
Ultrasound-assisted extraction	1:100	120	—	—	—	Song et al. (2025)
Ultrasound-assisted enzymatic extraction	1:25	100	73	450	13.67	Yang et al. (2025)

### 5.5 Effects of the extraction methods on the structural integrity of BRPs

Diverse isolation techniques may influence BRPs in two major ways: besides varying the final extraction recoveries, these approaches may also affect the intrinsic structural integrity of target polysaccharide molecules. It should be noted that robust direct experimental data revealing distinct degradation pathways that BRPs undergo during extraction remains insufficient at present. For this reason, the present section elaborates possible structural changes associated with diverse extraction parameters. Such analysis aims to interpret why BRPs isolated from different studies exhibit obvious discrepancies in the molecular weight distribution and overall structural features. Heat reflux extraction is easy to operate; yet, sustained heating under high temperature may trigger hydrolysis of glycosidic linkages, cutting long polysaccharide chains and lowering their average molecular mass. Acidic treatment helps release polysaccharide fractions bound to plant cell walls, whereas proton catalytic reactions may split glycosidic bonds, strip side-chains, and alter uronic-acid-containing regions. Alkaline conditions effectively extract wall-bound polysaccharides; yet, intensive alkali surroundings readily induce β-elimination, de-esterification, deacetylation, and conformational shifts of polysaccharide backbones. Ultrasonic-assisted extraction disrupts cell-wall structures and speeds mass transfer through cavitation effects; nonetheless, instantaneous micro-scale high-temperature, extreme local pressure, and mechanical shear force are capable of fragmenting polysaccharide chains. Enzymatic extraction proceeds under mild reaction environments, which degrades cell-wall composite substances to facilitate polysaccharide release. Even so, enzyme types, reaction pH, incubation temperature, and total enzymolysis duration may affect the completeness of separated polysaccharide fragments. In summary, optimizing BRPs extraction procedures cannot solely focus on maximizing recovery yields. Multiple vital indicators should be considered together, including retention of native molecular weight, branching architecture, uronic acid contents and stability of polysaccharide high-order spatial conformation.

## 6 Separation and purification methods

Separation and purification of BRPs generally consist of two stages: preliminary purification and fine purification. The following reasons are to ensure the purity and uniformity of the polysaccharide samples before structural characterization and biological activity analysis. Different purification methods will lead to structural changes in polysaccharide components, and thus, their biological activities will be different.

### 6.1 Preliminary purification

The first types of purification generally include ethanol precipitation, the Sevag method for de-proteinization, hydrogen peroxide-induced decolorization, and high-speed centrifugation (Dong and Yu, 2012; Song, 2006). Proteins, pigments, and other low-molecular-weight impurities are removed from the raw material at this time to reduce their adverse effects on subsequent detailed purification and structural analysis. The above will be the starting point for fine purification. The process parameters are adjusted based on the type and quantity of impurities in the crude polysaccharides, such as the number of de-proteinization cycles of the Sevag method and the concentration and duration of H_2_O_2_ decolorization, to prevent damage to the structure of the polysaccharides due to excessive treatment. For example, a relatively high concentration of H_2_O_2_ will oxidize the hydroxyl groups on the polysaccharides and break glycosidic bonds.

### 6.2 Fine purification

Column chromatography is a good way to obtain highly pure, uniform fractions of polysaccharides in the final step of purification. Chromatographic media that are frequently utilized can generally be divided into two types: DEAE-based ion-exchange materials (DEAE-Sephadex A-25 and DEAE-Sepharose) and gel filtration matrices (Sepharose CL-4B and Sepharose CL-6B). These two kinds have different ways of separation and apply in different places. Based on the above attributes, the purification of BRPs can be classified into two categories: separation methods and practical applications.

Studies have generally begun by using ion-exchange chromatography to achieve the first division according to the differences in the charge of the polysaccharides in the sense of separation strategy. DEAE-cellulose or DEAE-Sepharose columns are commonly used for this purpose, and stepwise or gradient elution with sodium chloride solutions is performed. After the first separation, gel-filtration chromatography is utilized to further divide the resulting fractions according to size differences, and structurally homogeneous polysaccharides are finally obtained. Ion-exchange chromatography and gel filtration have been used to purify BRPs to remove impurities and enhance the uniformity of polysaccharide fractions in a classical way.

In practical research applications, various purification systems have been utilized in conjunction with different raw materials and extraction methods to isolate and prepare multiple BR polysaccharides. For example, Liu et al. used crude polysaccharides obtained by water extraction as the starting material and sequentially applied DEAE-Sepharose and Sepharose 6 Fast Flow. This sequential approach—moving from ion-exchange to gel filtration—successfully isolated three structurally uniform fractions: BRP-W1, BRP-WA1, and BRP-WA2 (Liu et al., 2011). Following deproteinization and decolorization pre-treatment, Yang et al. used gradient elution on a DEAE-52 cellulose column to prepare four purified polysaccharide fractions (BRPS-0 to BRPS-3) (Yang et al., 2025). Similarly, Song utilized a combination of alkali extraction, preliminary purification, and sequential DEAE-Sephadex A-25 and Sepharose CL-4B column chromatography to obtain the homogeneous polysaccharide BRPS-IIIA (Song, 2006).

Several other studies have followed similar methodologies. Three polysaccharide fractions (BRPS-A1, BRPS-B1, and BRPS-B2) were isolated by Liu using a combination of DEAE-Sepharose and Sepharose CL-6B chromatography. A homogeneous polysaccharide, designated as BRT, was isolated by Wei et al., with its purity subsequently confirmed through both Sepharose CL-4B and DEAE-Sephadex A-25 chromatography (Wei et al., 2003). Chang G. et al. (1997) used the inflorescences of BR as experimental material to isolate and purify the water-soluble polysaccharide BWII *via* Sepharose CL-4B chromatography (Chang G. et al., 1997).

**FIGURE 3:**
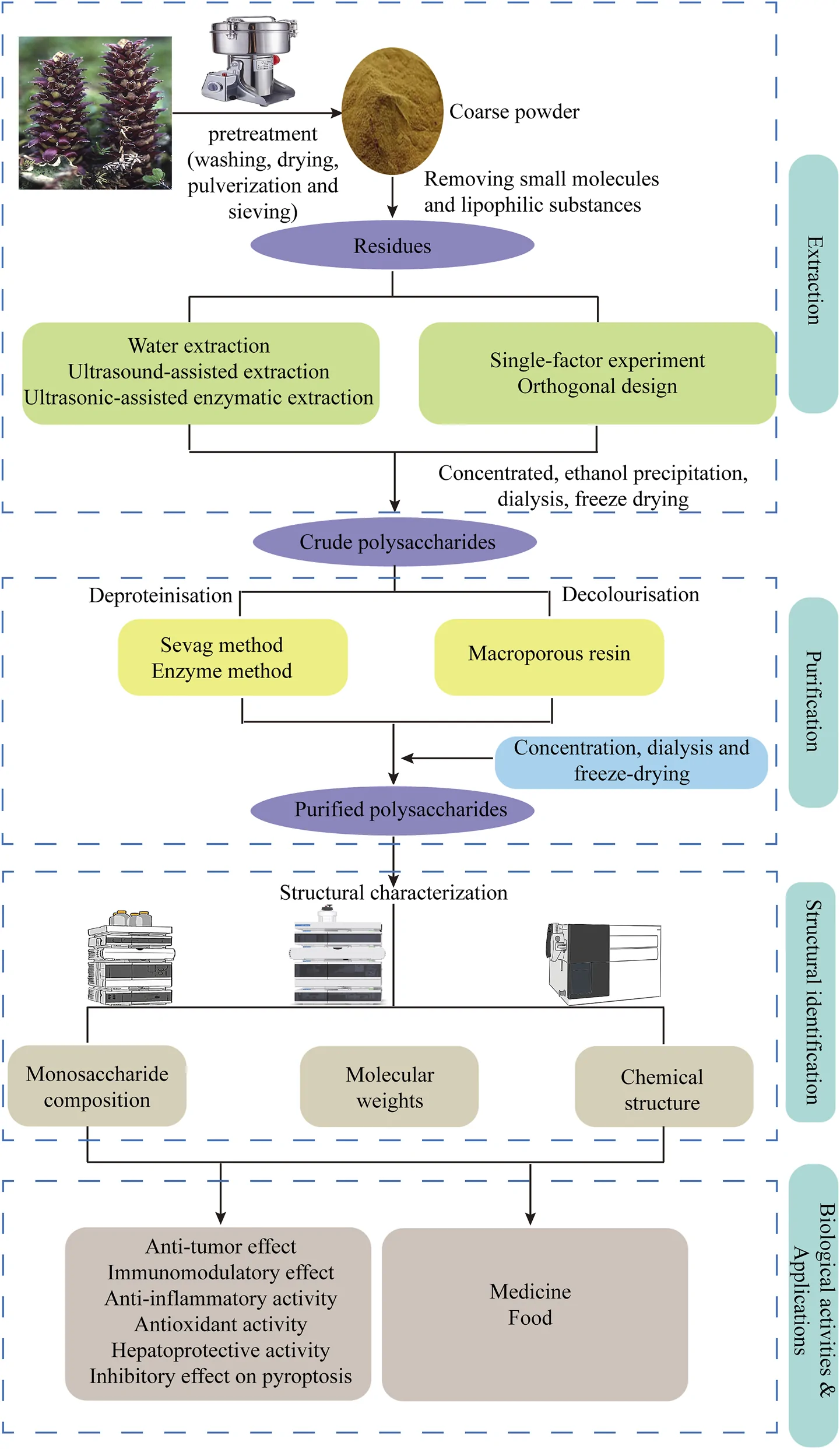
Schematic representation of the extraction, purification, structural characterization, and biological activities of *Boschniakia rossica* polysaccharides.

Through a systematic analysis of the key processing steps—including extraction, purification, deproteinization, and decolorization—a relatively well-established general workflow for the isolation and purification of BRPs has been developed. First, crude polysaccharides are prepared by washing, air-drying, and pulverizing the raw BR material into powder. To isolate the target fractions, crude extracts are obtained *via* hot water or ultrasound-assisted methods and refined through filtration. Subsequent addition of ethanol to the clarified filtrate serves to precipitate and enrich the polysaccharide content, ensuring a higher yield of the desired fractions. Crude BRPs are produced by freeze-drying the precipitate that is obtained. Subsequent purification steps focus on purging crude fractions of non-saccharide contaminants. This is achieved by leveraging the Sevag reagent to eliminate proteins and utilizing macroporous resin adsorption for pigment removal, thereby refining the polysaccharides and stripping out inorganic salts. To produce very pure polysaccharides, additional refinement processes including centrifugation and filtration are carried out before freeze-drying. Figure 3 outlines the general progression from BRPs extraction to biological evaluation, while Table 3 provides an overview of specific purification methods and structural features.

**TABLE 3:** Physicochemical properties, structural characteristics, and biological activities of purified polysaccharide fractions from *Boschniakia rossica*.

Fractions	Purification methods	Purification reagents	Analytical methods	Mw (Da)	Monosaccharide composition	Structural features	Biological activities	References
BRP-W1	DEAE Sepharose column chromatography and Sepharose 6B column chromatography	Proteinase Sevag reagent, distilled water, NaCl, absolute ethanol, acetone, and 95% ether	HPSEC, GC Congo red assay, phagocytosis assay, and Griess reaction ELISA	1.2 × 10^4^	Ara:Man:Gal:Glc:Xyl = 6:11:13:15:5	Non-triple-helical structure	Induces macrophage phagocytosis and immunostimulatory activity	Liu et al. (2011)
BRP-WA1	DEAE Sepharose column chromatography and Sepharose 6B column chromatography	Proteinase Sevag reagent, distilled water, NaCl, absolute ethanol, acetone, and 95% ether	HPSEC, GC Congo red assay, phagocytosis assay, and Griess reaction ELISA	3.0 × 10^4^	Man:Gal:Glc:GlcA = 8:20:50:21	Triple-helical structure	Induces macrophage phagocytosis and immunostimulatory activity	Liu et al. (2011)
BRP-WA2	DEAE Sepharose column chromatography and Sepharose 6B column chromatography	Proteinase Sevag reagent, distilled water, NaCl, absolute ethanol, acetone, and 95% ether	HPSEC, GC Congo red assay, phagocytosis assay, and Griess reaction ELISA	2.1 × 10^5^	Gal:Glc:GlcA = 23:52:24	Triple-helical structure	Induces macrophage phagocytosis and immunostimulatory activity	Liu et al. (2011)
BRPS-0	DEAE-52 cellulose column chromatography	Hydrogen peroxide, deionized water, and 0.1/0.3/0.5 mol/L NaCl solutions	GPC, FT-IR, ^1^H NMR,^13^C NMR, and PMP derivatization-HPLC	1,569	GalA:Glc:Gal: Ara = 0.352: 3.02:1.26:5.37	α- and β-pyranose configurations and characteristic polysaccharide absorption peaks (3,350, 2,930, 1,650, 1,410, and 1,040 cm^−1^) in FT-IR spectrum	Relatively stable antioxidant activity	Yang et al. (2025)
BRPS-1	DEAE-52 cellulose column chromatography	Hydrogen peroxide, deionized water, and 0.1/0.3/0.5 mol/L NaCl solutions	GPC, FT-IR, SEM, and phenol-sulfuric acid method	2,815	—	Homogeneous polysaccharide	Antioxidant activity	Yang et al. (2025)
BRPS-2	DEAE-52 cellulose column chromatography	Hydrogen peroxide, deionized water, and 0.1/0.3/0.5 mol/L NaCl solutions	GPC, FT-IR, SEM, and phenol-sulfuric acid method	4,572	—	Homogeneous polysaccharide	Antioxidant activity	Yang et al. (2025)
BRPS-3	DEAE-52 cellulose column chromatography	Hydrogen peroxide, deionized water, and 0.1/0.3/0.5 mol/L NaCl solutions	GPC, FT-IR, SEM, and phenol-sulfuric acid method	Heterogeneous (two fractions): 4,871 (peak 1) and 961(peak 2)	—	Heterogeneous polysaccharide and characteristic polysaccharide functional groups (2,940, 1,370, and 1,100 cm^−1^) in FT-IR spectrum	Antioxidant activity	Yang et al. (2025)
BRPS-IIIA	DEAE-Sephadex A-25 column chromatography and Sepharose CL-4B column chromatography	NaCl	GC	5 × 10^4^	Xyl:Gal:Man:Glc = 0.60:3.11:1.00:3.96	—	—	Song (2006)
BRPS-A1	Sevag DEAE-Sepharose column chromatography and Sepharose CL-6B column chromatography	Sevag reagent, absolute ethanol, acetone, and ether	GC, HPSEC, IR	1.2 × 10^4^	Ara:Man:Gal:Glc:Xyl = 13:22:26:30:10	—	*In vitro* antioxidant activity	Liu (2013)
BRPS-B1	Sevag DEAE-Sepharose column chromatography and Sepharose CL-6B column chromatography	Sevag reagent, absolute ethanol, acetone, and ether	GC, HPSEC, IR	3.0 × 10^4^	Man:Gal:Glc:GlcA = 8:20:50:21	—	*In vitro* antioxidant activity and anti-acute liver injury activity	Liu (2013)
BRPS-B2	Sevag DEAE-Sepharose column chromatography and Sepharose CL-6B column chromatography	Sevag reagent, absolute ethanol, acetone, and ether	GC, HPSEC, IR	2.1 × 10^5^	Gal:Glc:GlcA = 23:52:24	—	*In vitro* antioxidant activity and anti-acute liver injury activity	Liu (2013)
BRT	Sevag Sepharose CL-4B column chromatography and Sephadex A-25 column chromatography	Sevag reagent, deionized water, 90% ethanol, and absolute ethanol	PC, GC	5 × 10^4^	Xyl:Gal:Man:Glc = 0.61:3.12:1.00:3.97	The main chain is centered on (1→6)-Gal, (1→6)-Glc, and (1→4)-Glc; the side-chains contain (1→3)-Gal, (1→3)-Glc, (1→4)-Man, and (1→6)-Man	—	Wei et al. (2003); Wei et al. (2002)
BWII	Sevag Sepharose CL-4B column chromatography	Sevag reagent, distilled water, 85%/90%/95% ethanol, absolute ethanol, and ether	GC	3 × 10^4^	Fuc:Man:Gal:Glc:GlcA = 5.85:1.74:1.48:11.96:1	—	—	Chang et al. (1997)

## 7 Structural characteristics

The structure of polysaccharides affects how they are taken up in the body and, thus, determines their possible drug effects. Most of the characterized BRPs are heteropolysaccharides; their main structural features are advanced conformational properties, molecular weights (Mw), glycosidic linkage types, and monosaccharide composition. A series of structural characterization techniques, such as high-performance size-exclusion chromatography (HPSEC), high-performance liquid chromatography (HPLC), gas chromatography (GC), Fourier-transform infrared spectroscopy (FT-IR), and various column-based separations, have been used to elucidate the structures of BRP.

### 7.1 Monosaccharide composition

Galactose (Gal) and glucose (Glc) often make up the majority of the monosaccharides in BRPs (Yang and Yin, 1994). In addition, some fractions contain characteristic monosaccharides such as fucose (Fuc) and uronic acids, which together expand the structural multiplicity of BRPs, adding an extra layer of heterogeneity to the fractions. This difference in composition is considered the key structural basis for their various biological activities.

From the perspective of whole-plant polysaccharides, Liu et al. (2011) reported that the neutral polysaccharide fraction (BRP-W1) isolated from BR was composed of five monosaccharides: arabinose (Ara), mannose (Man), Gal, Glc, and xylose (Xyl) in a ratio of 6:11:13:15:5. In contrast, BRP-WA1 were mainly composed of Man, Gal, Glc, and glucuronic acid (GlcA) in an 8:20:50:21 ratio, while BRP-WA2 comprised only of Gal, Glc, and GlcA in a ratio of 23:52:24, with no Ara or Xyl detected. Among them, the presence of uronic acid is the main reason for their acidity.

The monosaccharide profiles of the polysaccharides in these tissues also differ significantly depending on their place of origin. For example, Ara, Xyl, Gal, Man, and Glc are water-soluble crude polysaccharides extracted from rhizomes, and their typical molar ratios are 0.98:1.00:2.38:1.28:10.00. Inflorescence-derived polysaccharides, on the other hand, are characterized by the inclusion of Fuc and galacturonic acid (GalA) and have a complex Fuc:Xyl:Glc:Gal:Ara:GalA assembly of 2.60:5.75:21.88:21.0:1.00:3.48 (Chang et al., 1999a). Fuc is an index factor that may be responsible for the particular treatment efficacy of fungi and help regulate the immune system or reduce inflammation.

Extraction, separation, and purification may also reduce the amount of monosaccharides in BRPs. Purified polysaccharides generally have a more certain composition and significantly less impurity interference. Wang et al. prepared a homogeneous BRP (purity 96.9%) that showed no detectable protein or nucleic acid signals in the Bradford assay or UV spectra (260/280 nm) (Wang et al., 2012). Similarly, the polysaccharide fraction (BRPS-IIIA) isolated from rhizomes by Song (2006) is composed of Xyl, Gal, Man, and Glc in a ratio of 0.60:3.11:1.00:3.96, and it is a neutral heteropolysaccharide. Chang (Chang G. et al., 1997) also pointed out that the polysaccharide obtained from inflorescences (BWII) contains Fuc, Glc, GlcA, Gal, Man, and Ara, and Fuc is one of these characteristic monosaccharides.

In addition, early studies of BR from the Changbai Mountain area have confirmed that its polysaccharides are all heteropolysaccharides composed of multiple monosaccharides rather than homopolysaccharides (polymers of a single monosaccharide) (Li et al., 1987).

### 7.2 Molecular weight

A structural feature of BRP is a relatively high molecular weight (Mw), and this may lead to poor solubility and a higher-order conformation in the body. Several studies have found that the MWs of BRPs vary greatly with different parts of the plant and other conditions. Gel filtration chromatography and HPAE fractionation can be used to determine the size distribution and monodispersity of the sample.

After several times of purifying by ion-exchange and size-exclusion chromatography, the resulting BRPs have been relatively uniform in size and have a narrower Mw distribution. For example, Liu et al. (2011) separated three different polysaccharide fractions (BRP-W1, BRP-WA1, and BRP-WA2) from the aqueous extract of BR by DEAE-Sepharose and Sepharose 6 Fast Flow. The average molecular masses of the three low- to high-molecular-weight fractions separated by HPSEC analysis were 1.2 × 10^4^ Da, 3.0 × 10^4^ Da, and 2.1 × 10^5^ Da, respectively. The single, clear, and symmetric elution peak of each fraction was very narrow and, therefore, highly uniform. Chang and others purified a water-soluble isolate (BWII) from BR inflorescences in the same way and determined that it was a heteropolysaccharide with a relatively low average molecular weight of 3.0 × 10^4^ Da (Chang G. et al., 1997).

In addition to purification-induced differences, other reasons for the shift in Mw have been identified. Polysaccharides extracted from the rhizome and stem (BRT) had a relatively high molecular weight of approximately 5.0 × 10^4^ Da and were determined by gel filtration chromatography (Wei et al., 2003). The total saccharide content of this fraction was relatively high (94%), and the degree of branching was low; the main backbone components were glucose residues.

Marked disparities in molecular weight profiles of BRPs have been documented across independent research reports. Such numerical deviations should not be simply interpreted as inconsistent experimental outcomes; instead, they may arise from multiple material-related, processing-related, and analytical factors.

First, different segments of harvested plants, various areas in nature, and different stages of growth for raw materials can all produce different primary polysaccharide contents. Second, the extraction temperature and time, pH value, solid-to-liquid ratio, ultrasonic power, and enzymatic hydrolysis conditions, among other processing parameters, will affect the release, degradation, and recovery of carbohydrate polymers. Given that the general degradation characteristics of extracted polysaccharides are shown above, extended high-temperature treatment, extreme acid–base conditions, and multiple ultrasonic treatments can cause glycosidic bond scission and a reduction in the apparent molecular weight of the final product. Third, different purification workflows will also change the scope of measurable molecular weight. Crude polysaccharide extracts, de-proteinized crude products, ion-exchange separated fractions, and gel filtration purified isolates cannot be directly compared because they are all different polysaccharide populations separated by electric charge or molecular mass. Finally, there are also detection-related differences in the last step, such as different methods of molecular weight measurement, chromatographic column specifications, selection of reference standards, and data processing algorithms.

Therefore, follow-up research on BRPs is recommended to provide all-encompassing supporting data along with molecular weight values, such as the raw material background, the entire extraction and purification workflow, detailed detection protocols, and a full molecular weight distribution profile, rather than just reporting a single average molecular weight figure.

### 7.3 Glycosidic linkage types

FT-IR spectroscopy can be used to obtain some structural and functional group information about BRPs relatively quickly. To obtain specific structural information of glycosidic linkage patterns, methylation analysis and Smith degradation can be used in conjunction with periodate oxidation to prepare GC-detectable partial acid hydrolyzates (Gao et al., 2026). Different arrangements of glycosidic bonds have different structures and functions for polysaccharides.

**FIGURE 4:**
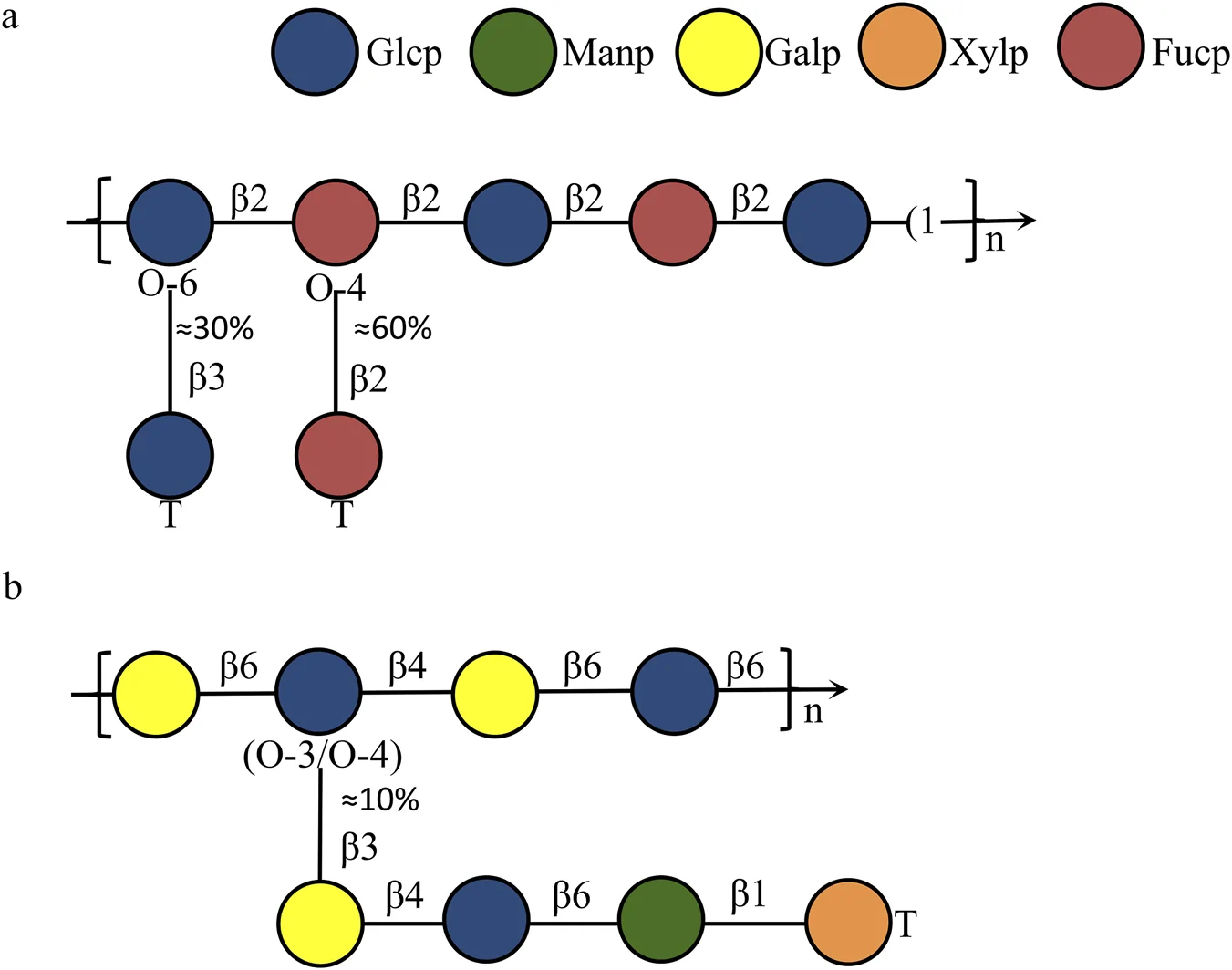
Proposed molecular structures of representative polysaccharides from *Boschniakia rossica*: **(a)** BJIIa and **(b)** BRT.

Chang et al. (1997b) reported that the FT-IR spectrum of BJIIa exhibited a characteristic absorption peak at 890 cm^−1^, indicating the presence of β-glycosidic linkages. Additional absorption bands at 1,076, 1,273, 1,403, 1,623, and 3,200 cm^−1^–3,400 cm^−1^ further confirmed the typical polysaccharide structural features. Subsequent GC and methylation analyses revealed that BJIIa is a highly branched neutral heteropolysaccharide. Its backbone consists mainly of (1→2)-linked Fuc and Glc residues, with repeating units composed of approximately 10 monosaccharide residues. The side-chains include (1→3)-linked Glc and (1→2)-linked Fuc, with branching occurring at the O-6 position of Glc and the O-4 position of Fuc and branching rates of approximately 30% and 60%, respectively (Figure 4a) (Chang, 2000). Similarly, Wei et al. (2002) performed enzymatic hydrolysis and methylation analysis and demonstrated that the polysaccharide BRT predominantly contains β-type linkages, including terminal (1→)-linked residues (Xyl and Glc), along with (1→3), (1→4), (1→6), and branched (1→3, 4) linkages. Its backbone is primarily composed of (1→6)-linked Gal and (1→4)/(1→6)-linked Glc, while the side-chains consist of Gal, Glc, and Man residues linked *via* (1→3), (1→4), and (1→6) bonds, forming a relatively low-branched structure with approximately one branching point per 10 backbone residues (Figure 4b). In addition, alkali-extracted water-soluble polysaccharides from the inflorescence have been reported to possess a backbone mainly composed of (1→2)-linked Glc and Fuc residues, with a lot of branching, where three of every four backbone residues are substituted with side-chains. A small proportion of Glc residues are located at the terminal positions of the structure (Chang et al., 1999b).

### 7.4 Higher-order structure and glycoconjugate features

Some BRPs exist as glycoprotein–polysaccharide component, and current research has been focused largely on the glycoprotein fractions derived from alkali-extracted polysaccharides isolated from BR inflorescences. As reported by Lü et al., the protein moiety associated with these water-soluble polysaccharides represents a binding protein with an approximate molecular weight of 40 kDa. This protein is thought to covalently bind either to the terminal regions of the polysaccharide backbone or to the non-reducing ends of (1→6)-linked Glc residues and (1→4)-linked Fuc branches (Lü, 2014).

The spatial conformation of BRPs in aqueous solution was evaluated using the Congo red assay. The acidic polysaccharides, BRP-WA1 and BRP-WA2, exhibited a characteristic redshift in the maximum absorption wavelength under 0.3 M NaOH, indicating the presence of a triple-helix conformation in solution. This feature may represent a characteristic higher-order structural property of these acidic polysaccharide fractions. In contrast, no obvious shift in the maximum absorption wavelength was observed for the neutral polysaccharide BRP-W1, indicating the absence of detectable triple-helix structures under the tested conditions (Liu et al., 2011). Such conformational differences may be associated with variations in immunological activity between acidic and neutral BRPs; however, further studies are needed to establish a definitive structure–activity relationship.

### 7.5 Chemical antioxidant capacity as an analytical property

Several studies have evaluated the chemical antioxidant capacity of BRPs using DPPH, ABTS, superoxide anion, hydroxyl radical scavenging, and reducing power assays. It is worth stressing that a series of chemical analytical tests including DPPH radical scavenging, ABTS inhibition, hydroxyl radical removal, and reducing capacity evaluation mainly reflect the free radical elimination efficiency and reducing features of BRPs. Li et al. further evaluated the total antioxidant capacity and determined the MDA content using the thiobarbituric acid (TBA) assay (Li et al. 2011). Such physicochemical test results are not sufficient to serve as direct proof of pharmacologically relevant antioxidant effect in living biological environments.

## 8 Structure–activity relationships

Within the realm of polysaccharides, structure–activity relationships signify the intrinsic link between their physicochemical properties, primary structures, higher-order structures, and pharmacological activities (Shang et al., 2019). In the case of BRPs, bioactivity is governed by diverse structural attributes, specifically the monosaccharide profile, linkage modalities, molar mass, and higher-order conformations.

Jin et al. reported that a neutral BRP fraction with a Mw of approximately 4.9 × 10^4^ Da exhibited significant protective effects against H_2_O_2_-induced damage in HL-7702 human hepatocytes. The underlying mechanism is associated with the apoptosis prevention and modulation of the MAPK signaling pathway (Jin et al., 2021). In another study, four polysaccharide fractions (BRPS-0, BRPS-1, BRPS-2, and BRPS-3) were isolated *via* ultrasound-aided enzymatic means, yielding molar masses of 1,569, 2,815, and 4,572 Da, respectively, for the first three samples. Distinct from the others, BRPS-3 featured a more intricate profile with two elution peaks (4,871 and 961 Da). Among them, BRPS-0 exhibited relatively stable antioxidant activity, which stems from its low molecular weight, relatively high phenolic and carbohydrate contents, and specific structural features such as α/β-pyranose configurations and a partially crystalline microstructure (Yang et al., 2025). Regarding immunomodulatory activity, Liu et al. elucidated that BRP-W1 was devoid of uronic acids, whereas BRP-WA1 and BRP-WA2 contained 23.2% and 25.3% uronic acids, respectively. Compared with the neutral fraction, the two acidic polysaccharides had significantly stronger effects on macrophage phagocytosis, nitric oxide (NO) production, and tumor necrosis factor-α (TNF-α) secretion. Congo red analysis indicated that BRP-WA1 and BRP-WA2 adopt triple-helix conformations in solution, whereas BRP-W1 does not exhibit such structural features under the tested conditions (Liu et al., 2011). These results indicate that both the presence of uronic acids and higher-order conformations may contribute to the enhanced immunological activity.

Structural modification is considered a viable strategy for enhancing the biological activities of polysaccharides. Although studies on BRP modification are still limited, their inherent hydroxyl and carboxyl functionalities provide potential sites for derivatization. Chemical modifications such as sulfation, acetylation, and carboxymethylation are expected to alter the physicochemical properties and spatial conformations of BRPs, thus enhancing their antioxidant and immunomodulatory activities. Therefore, systematic investigations into the structure–activity relationships of BRPs through controlled structural modification may provide promising strategies for structural optimization and potential pharmaceutical development.

Although the above studies have been conducted, the structure–activity relationships for BRPs are still unknown. Polysaccharides have diverse structures; the extraction methods vary, and standardization of characterization technology is absent; thus, direct comparison is not feasible. Therefore, some new, organized research will be carried out to explore advanced structural analysis and bioactivity.

## 9 Pharmacological effects

A large volume of research concerning the reported experimental bioactivities of BRPs has been published; yet, the reliability of available evidence varies widely. The most robust research data currently come from phytochemical profiling, cellular mechanistic exploration, and *in vivo* animal trials, while rigorous human clinical verification remains scarce. For this reason, the various biological activities elaborated in the following sections cannot be deemed equally definitive. Several hypothesized functional mechanisms are only backed by *in vitro* cellular tests. Though certain pharmacological effects have been validated *via* animal models, they lack confirmation from well-controlled human clinical trials. When evaluating the therapeutic prospects of BRPs, it is necessary to distinguish preliminary preclinical findings from evidence suitable for clinical translation or clinical use. Table 4 summarizes reported BRP-related bioactivity studies, including the test materials, experimental models, dose/duration, controls, major findings, key limitations, and references.

**TABLE 4:** Reported pharmacological effects and proposed mechanisms of BRPs. This table summarizes the pharmacological activities of isolated constituents and crude extracts of BRPs across *in vitro* and *in vivo* models, organized by the test material, pharmacological activity, experimental model, dose/duration, controls, major findings, key limitations, and references.

Pharmacological activity	Test material	Experimental model	Dose/duration	Controls	Major findings	Key limitations	Reference(s)
Tumor cell growth-inhibitory and pro-apoptotic effects	BRPs	*In vitro* anti-tumor model using human laryngeal squamous carcinoma Hep2 cells HepG2 hepatocellular carcinoma cells S180 sarcoma-bearing mice	200 μg/mL for cell-cycle analysis 100 μg/mL–400 μg/mL for 24 h, 200 μg/mL for cell-cycle analysis BRP 50 mg/kg/day–200 mg/kg/day orally for 10 days, 5-FU 25 mg/kg with or without BRP.	5-FU/BRP alone, and combination groups	Promoted the cleavage of pro-caspase-3, pro-caspase-8, and pro-caspase-9; upregulated the expression of death receptor DR5 and Bax; and downregulated Bcl-2 expression Inhibited the proliferation and migration of Hep-2 cells and downregulated the expression of PD-1/PD-L1; reduced the migration and invasion abilities of HepG2 cells; regulated HIF-1α expression; and inhibited EMT progression Inhibited the growth of S180 sarcoma when combined with 5-FU, stimulating lymphocytes proliferation, increasing NK cell cytotoxicity, enhancing serum IL-2 and TFN-γ secretion, along with augmenting CD4^+^ and CD8^+^ spleen T lymphocytes subsets	Single cell line; no normal-cell, *in vivo*, or target-rescue validation; *in vitro* only, no normal-cell or positive-drug control; one short-term transplant model, no synergy analysis, survival, pharmacokinetic, or comprehensive safety data	Wang et al. (2014), Zhang et al. (2014), Cui et al. (2021), Hua et al. (2021), and Wang et al. (2012)
Immunomodulatory effects	BRPs	*In vitro* culture model of mouse splenocytes BALB/C(H-2) mice; *in vitro* macrophage activation model.	12.5 mg/kg/day–200 mg/kg/day intraperitoneally for 7 days, 25 mg/L–400 mg/L *in vitro* 50 μg/mL–400 μg/mL for 24h–48 h	Saline or RPMI-1640 controls, LPS stimulation, no positive drug Medium and LPS controls, endotoxin-negative	Upregulated IL-2 and enhanced immune capacity of mice Increased the number of specific antibody-producing cells against sheep red blood cells in mouse spleen Enhanced phagocytic activity and elevated NO and TNF-α	No cell-subset, pathway, or human validation No *in vivo* validation	Liang et al. (2009), Zhang et al. (2000), and Liu et al. (2011)
Anti-inflammatory effects	Extraction of BRPs by conventional ethanol precipitation	LPS-induced inflammatory model in THP-1 macrophages *In vitro* model of inflammasome activation in mouse J774A.1 macrophages induced by LPS combined with ATP.	BRPs 25 mg/L–100 mg/L for 24 h (screen: 12.5 mg/L–200 mg/L), LPS 100 μg/L for 1 h BRPs 25 mg/L–100 mg/L for 24 h, LPS 200 μg/L for 12 h, ATP 2 mmol/L for 30 min	Blank/model, BRPS, inhibitor, and combination groups; no positive drug Normal/model and inhibitor ± BRPS groups; no positive drug	Reduced ROS and the protein levels of NLRP3, caspase-1, GSDMD-N, and IL-1β Reduced LDH release and alleviated cell membrane damage; downregulated NLRP3, mature IL-1β, and GSDMD-N protein levels, while upregulating pro-caspase-1 expression	No primary-cell or *in vivo* validation Single mouse cell line; no primary-cell or *in vivo* validation	Ma et al. (2024) and Zhang et al. (2024)
Antioxidant effects	Extraction of BRPs *via* conventional method	H_2_O_2_-triggered oxidative hemolysis injury model of erythrocytes *in vitro*	Up to 5 mg/mL, 10-min pretreatment followed by 1-h oxidation	Normal and oxidant-model controls, no positive antioxidant	Reduced MDA level and exerted *in vitro* anti-lipid peroxidation activity Anti-lipid peroxidation and red blood cell protection	No *in vivo* disease model, pharmacokinetics, positive control, or target validation	Jin A. H. et al. (2011)
Hepatoprotective effects	Extraction of BRPs *via* conventional method Hot-water extracted BRPs	*In vitro* model of H_2_O_2_-induced oxidative injury in HL-7702 human hepatocytes H_2_O_2_-triggered oxidative stress damage model of HL-7702 hepatocytes *In vivo* model of CCl_4_-induced acute liver injury in mice	BRP 50mg/L–100 mg/L for 12 h, H_2_O_2_ 300 μmol/L for 6 h (safety screen: 0 mg/L–400 mg/L) BRPs 100 mg/kg/day–200 mg/kg/day orally for 7 days, silymarin 50 mg/kg/day, CCl4 80 mg/kg intraperitoneally	Normal/model and low/high BRP groups Normal, CCl4 model, silymarin, and BRPS-alone groups	BRP at 50 and 100 mg/L improved cell survival, suppressed LDH release, and MDA accumulation to relieve hepatic oxidative injury 50 mg/L BRPs significantly increased hepatocyte viability; reduced the release of ALT, AST, and LDH; elevated SOD and GSH levels; and decreased MDA content Downregulated ALT, AST, ALP, and MDA, while upregulating GSH and SOD.	No primary-cell or *in vivo* validation Well-controlled animal evidence, but prophylactic and limited to one acute model	Jin et al. (2021), Yin et al. (2017), and Quan et al. (2013)
Anti-aging	Crude ethanol extract of BR	D-galactose-induced sub-acute aging rat model	D-galactose 48 mg/kg/day subcutaneously, extract 100 mg/kg/day–200 mg/kg/day orally for 40 days; ginsenosides 100 mg/kg/day	Normal, model, and ginsenoside controls	Elevated SOD activity, reduced MDA content, and inhibited MAO activity	No behavioral or long-term outcomes	Piao et al. (2003) and Yang et al. (2024)
Cardioprotective	Extraction of BRPS by ethanol precipitation	H/R-induced injury model in H9c2 rat cardiomyocytes	BRPs 25 mg/L–100 mg/L for 24 h, H/R for 3 h/4 h	Normal/model, dose, and miRNA controls; no positive drug	Reduced MDA level, miR-302a-3p expression, cell apoptosis rate, and Bax expression; elevated SOD, GSH-Px, and Bcl-2 levels	Single H9c2 model; no primary-cell, *in vivo*, or pharmacokinetic validation	Chen S. K. et al. (2023)

BRPs have many types of biological activities, such as tumor-cell growth-inhibitory and pro-apoptotic effects (Yin et al., 2000), immunomodulation, antioxidant effects (Liu et al., 2020), anti-inflammatory activities (Ma et al., 2024), hepatoprotection (Jin et al., 2017), and anti-aging and cardioprotective effects (Liu, 2022). BRPs have also been shown to be relatively non-toxic, and no mutagenic effects have been observed under the conditions of the experiment (Fei et al., 2008).

### 9.1 Tumor-cell growth-inhibitory and pro-apoptotic effects

Most of the available research concerning BRPs relies on *in vitro* tumor-cell systems (Hep-2 and HepG2 cell lines, for instance) and animal models to document how this polysaccharide substance modulates malignant cell proliferation, migratory capacity, invasive behavior, and apoptotic progression. On this basis, this section only defines the above biological responses as tumor-cell growth retardation and pro-apoptotic properties demonstrated within experimental research frameworks rather than directly equating these preclinical findings with clinically effective antitumor therapy.

Wang et al. found that BRPs caused cell-cycle arrest at the G0/G1 phase and promoted apoptosis in Hep-2 laryngeal carcinoma cells. These effects were associated with the activation of both the mitochondrial-dependent intrinsic apoptotic pathway and the DR5-related extrinsic apoptotic pathway. The treatment also exerted regulatory Bcl-2 and Bax expression and triggered the caspase-3/-8/-9 cascade, thereby contributing to tumor-cell growth-inhibition and apoptosis (Wang et al., 2014; Zhang et al., 2014). Moreover, BRPs downregulated the mRNA and protein expression levels of programmed cell-death protein 1 (PD-1) and its ligand, PD-L1, thereby inhibiting the proliferation, colony formation, and migration of Hep-2 cells (Cui et al., 2021).

Hua et al. found that BRPs induce apoptosis in HepG2 cells and inhibit their migration and invasion in a hepatocellular carcinoma model. These effects may be associated with the suppression of epithelial–mesenchymal transition (EMT) and the concomitant reduction of hypoxia-inducible factor-1α (HIF-1α) expression (Hua et al., 2021). Furthermore, studies by Wu and Xue et al. indicated that rhizoma-derived BR polysaccharides alleviate oxidative damage by minimizing malondialdehyde (MDA) levels and augmenting the enzymatic activity of superoxide dismutase (SOD) activity. They also improve antitumor immune responses by elevating serum tumor necrosis factor-α (TNF-α) levels, thereby exerting both antitumor and hepatoprotective effects in the early-to-intermediate stages of hepatocellular carcinoma (Wu et al., 2011; Xue et al., 2011).

**FIGURE 5:**
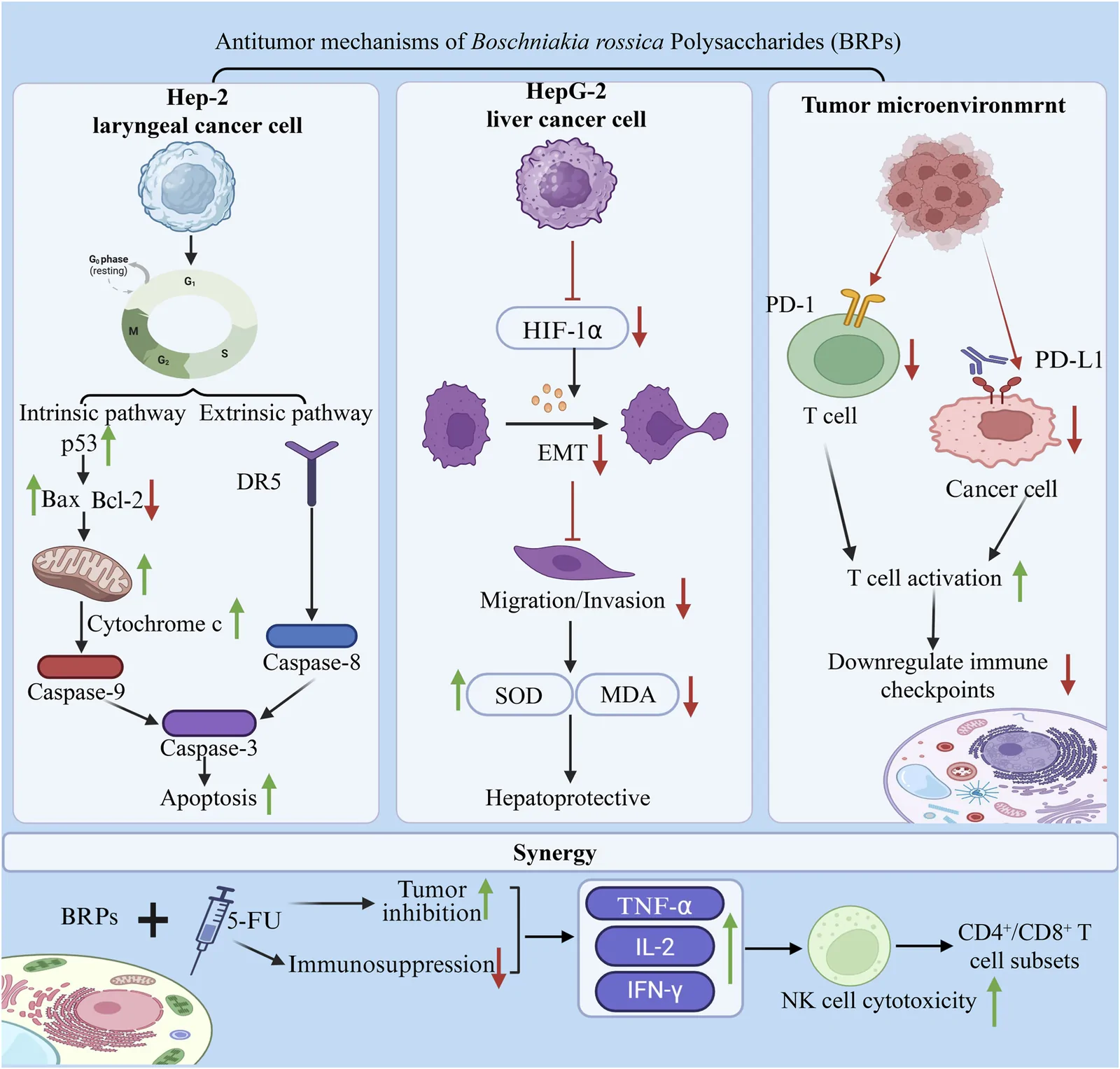
Antitumor mechanisms of BRPs (Created with BioRender.com).

BRPs have suppressed the division of tumor cells and shown some synergy with chemotherapy. Wang and others have shown that BRP (50 mg/kg–200 mg/kg) inhibits the growth of tumors in an S180 mouse model in a dose-dependent manner, and the maximum tumor inhibition achieved by BRP alone was 40.9%. The ratio of tumor inhibition by 5-FU was 0.641. BRP also alleviated 5-FU-induced immunosuppression by promoting the proliferation of splenic lymphocytes, enhancing natural killer (NK) cell cytotoxicity, increasing serum interleukin-2 (IL-2) and interferon-γ (TFN-γ) levels, and modulating CD4^+^ and CD8^+^ T-cell subsets; thus, it achieved stronger anti-tumor effects in the S180 mouse model when combined with 5-FU (Wang et al., 2012). In short, BRPs have shown an anti-tumor effect in the S180 mouse model by means such as inducing apoptosis of tumor cells, inhibiting their proliferation and metastasis, and modulating the immune system. Figure 5 shows the reported tumor-related effects.

Collectively, the accumulated experimental results show that BRPs can inhibit the proliferation of tumor cells and trigger apoptotic programs in a laboratory setting; however, almost all supporting evidence has been obtained through cell-level tests and animal model studies. Most of the previous studies have used crude polysaccharide extracts or only partially characterized polysaccharide fractions; furthermore, these studies have not utilized a unified dosing protocol, a standard positive control group, or a human clinical validation design. Given the above circumstances, only the current experimental data are available to support the inference of an inhibitory effect on tumor cell proliferation and a pro-apoptotic function of BRPs. Future studies should use BRP preparations with clear structural features and standardized quality control indicators. Dose–response experiments, models of the tumor microenvironment, and *in vivo* pharmacodynamic assays should be combined in the future to explore the therapeutic effects of BRPs in more depth.

### 9.2 Immunomodulatory effects

**FIGURE 6:**
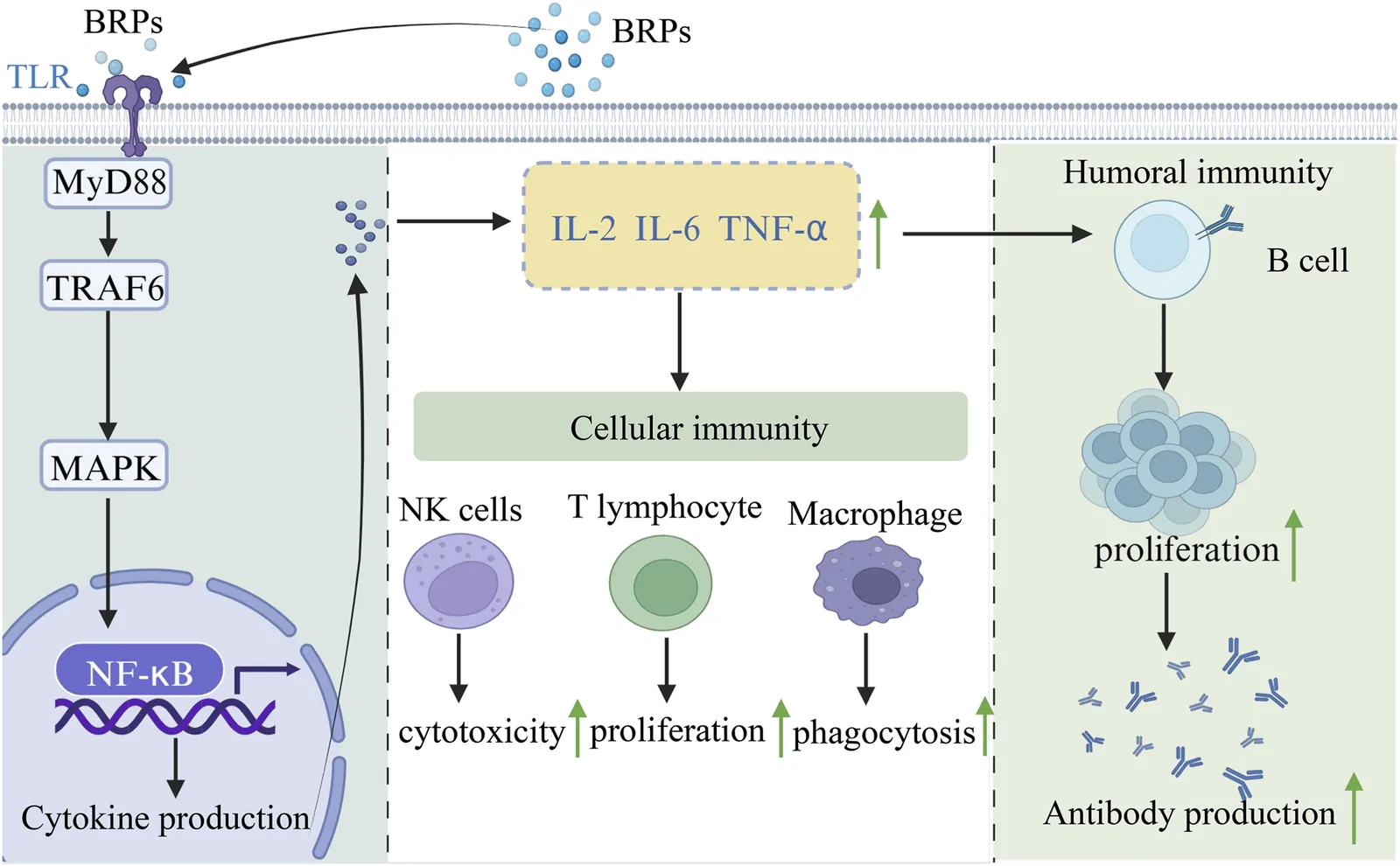
Immunomodulatory mechanisms of BRPs (Created with BioRender.com).

Immunomodulatory activity is among the most extensively studied pharmacological activities of BRPs. BRPs have been shown to enhance immune responses by activating the TLR/MyD88 signaling pathway and regulating the secretion of key cytokines. As IL-2 is indispensable for the proliferation and function of T lymphocytes, its upregulation likely underpins the immune-enhancing properties exhibited by BRPs (Liang et al., 2009). Zhang et al. demonstrated that BRPs promote IL-2 secretion by mouse splenocytes, thereby enhancing immune function (Zhang et al., 2000). In addition to cytokine modulation, BRPs can influence both humoral and cellular immunity. In terms of humoral immunity, BRPs have been shown to promote antibody-mediated immune responses by elevating the population of splenic antibody-secreting cells and B lymphocyte proliferation in response to lipopolysaccharide stimulation (Zhang L. et al., 2016; Zhang et al., 1999). With respect to cellular immunity, BRPs have been shown to activate macrophages, leading to increased phagocytic activity and triggering the release of pro-inflammatory mediators, including NO and TNF-α (Liu et al., 2011). Furthermore, BRPs dose-dependently boost T-lymphocyte transformation, natural killer (NK)-cell activity, and macrophage phagocytosis in H22 tumor-bearing mice models (Hou et al., 2007). Collectively, BRPs exert potent anti-tumor immunomodulatory capacity by promoting the secretion of cytokines, strengthening both humoral and cellular immune responses, and activating immune effector cells, as illustrated in Figure 6.

Experimental outcomes from existing studies reveal that BRPs are able to modulate the functional status of macrophages, T lymphocytes, and natural killer cells, alongside multiple immune cytokines such as IL-2 and TNF-α, which reflects the promising capacity of these polysaccharides to regulate immune functions. Even so, most published works only described the fluctuation of immune-related indicators without further mechanistic exploration. Researchers have not yet identified the exact membrane receptors on immune cells that bind to BRPs, nor have they clarified the earliest molecular events initiating the subsequent immune signaling cascades. Follow-up studies need to focus on validating the involvement of several pattern-recognition receptors, including TLR4, Dectin-1, and mannose receptors, in the immunomodulatory effects induced by BRPs.

### 9.3 Anti-inflammatory effects

**FIGURE 7:**
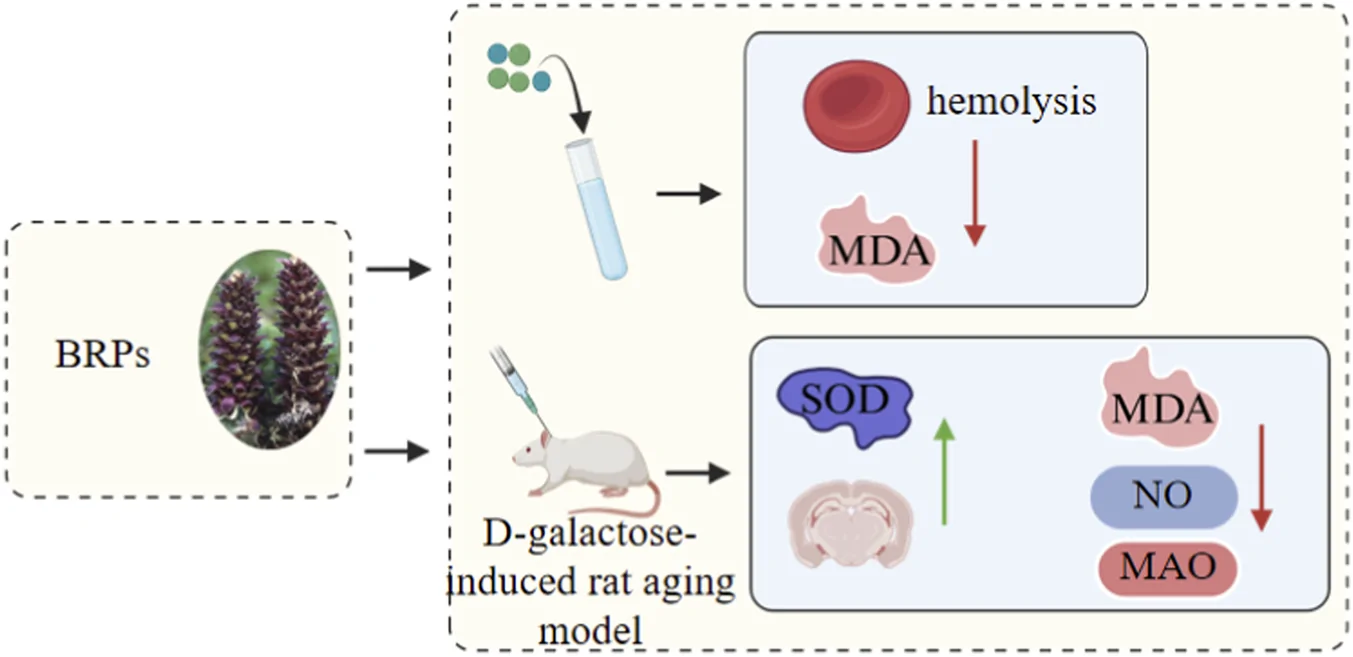
Anti-inflammatory mechanisms of BRPs (Created with BioRender.com).

The anti-inflammatory effects of BRPs are associated with their ability to influence various signaling pathways and limit the release of pro-inflammatory factors. Ma et al. reported that BRPs protect against lipopolysaccharide (LPS)-induced inflammatory injury in THP-1 macrophages by intervening in the phosphorylation and activation of NF-κB and MAPK (p38/ERK/JNK) pathways. These effects are associated with reduced reactive oxygen species (ROS) production, attenuation of mitochondrial damage and cell membrane disruption, suppression of NLRP3 inflammasome activation, caspase-1 cleavage, and the N-terminal fragment of gasdermin D (GSDMD-N)-mediated pyroptosis, along with decreased release of pro-inflammatory mediators, including interleukin-1β (IL-1β), IL-6, and TNF-α (Ma et al., 2024). Zhang et al. further demonstrated that polysaccharides from BR exert potent anti-inflammatory properties mainly through suppression of the NF-κB and MAPK signaling pathways. This regulatory action subsequently thwarts the triggering of the NLRP3 inflammasome and halts pyroptosis driven by gasdermin D (GSDMD) (Zhang et al., 2024). Fei et al. utilized xylene-triggered mouse ear edema, dextran-elicited rat paw edema, and a peritoneal leukocyte exudation model and demonstrated that BRP exerts significant acute and sub-acute anti-inflammatory effects. These outcomes are achieved through the dose-dependent stabilization of capillary permeability, alongside the mitigation of leukocyte and neutrophil chemotaxis and a reduction in the secretion of pro-inflammatory factors (Fei et al., 2014). To conclude, the multi-targeted anti-inflammatory profile of BRPs underscores their promise as natural candidates for the intervention and relief of inflammation. Figure 7 shows the anti-inflammatory role of BRPs.

Current research concerning the anti-inflammatory effects exerted by BRPs predominantly focuses on the dynamic alterations of downstream signaling pathways involving NF-κB, MAPK, NLRP3, and GSDMD. Such experimental data can deliver tentative mechanistic evidence to interpret the inflammatory modulatory capacity of BRPs; yet, few studies have elaborated the specific molecular processes whereby host cells recognize these polysaccharide components and activate relevant regulatory signaling cascades. To fully uncover the upstream molecular targets and intact regulatory mechanism network of BRPs, follow-up research can integrate multiple research techniques including receptor blocking assays, gene knockdown technology, and signaling pathway inhibitor intervention.

### 9.4 Antioxidant effects

**FIGURE 8:**
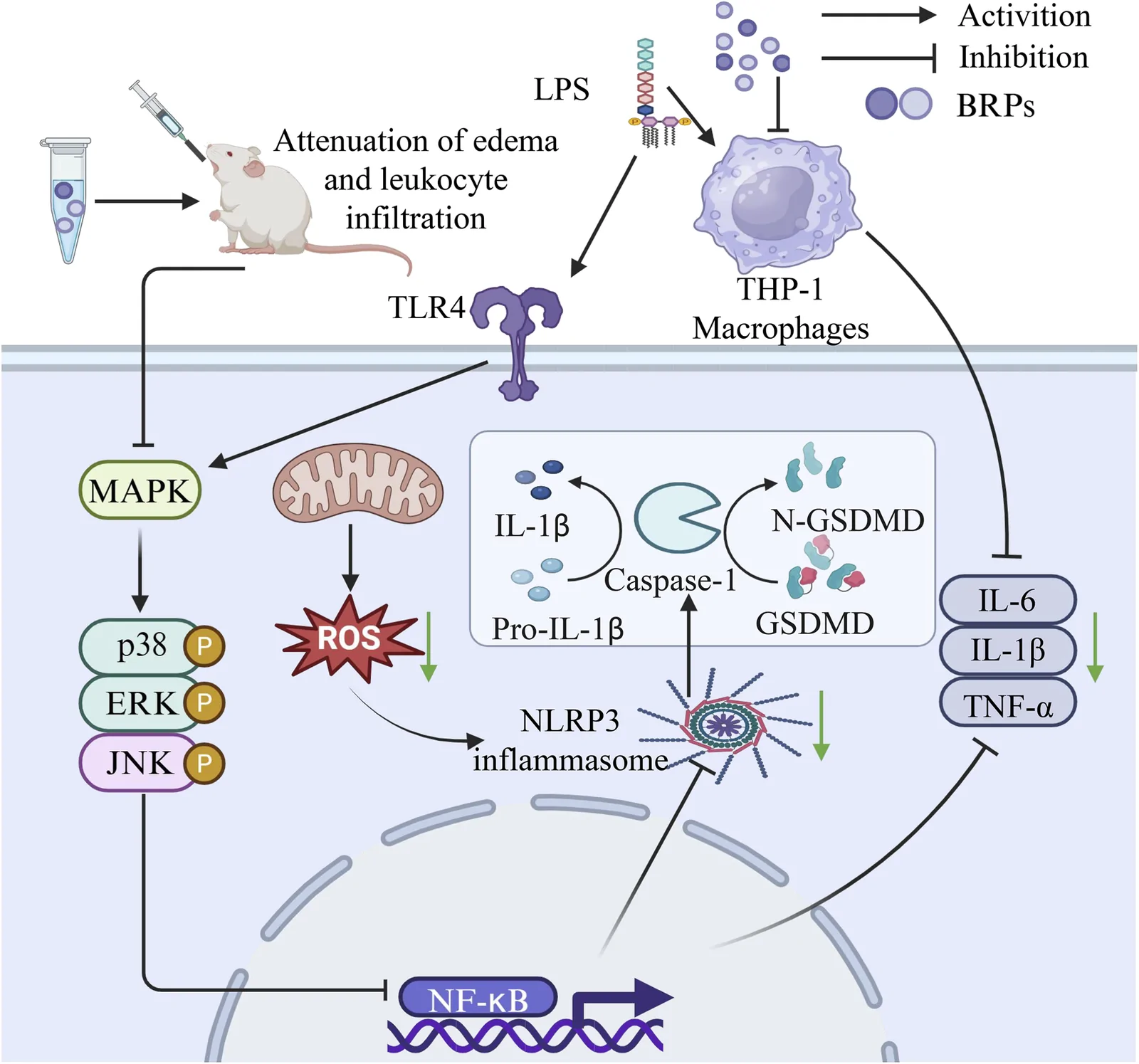
Antioxidant mechanisms of BRPs (Created with BioRender.com).

Many natural plant-derived polysaccharides serve as potent antioxidants that intervene in oxidative damage by eliminating reactive oxygen species and suppressing lipid peroxidation, thereby helping to prevent various chronic ailments mediated by oxidative stress (Liu et al., 2024). *In vivo*, these polysaccharides increase SOD activity, reduce MDA and NO levels, and inhibit lipid peroxidation, thereby alleviating oxidative stress (Liu, 2013). The results demonstrated that BRPs dose-dependently enhanced total antioxidant capacity. These effects are driven by the neutralization of reactive oxygen species and the thwarting of lipid peroxidation chain reactions, which collectively lead to the mitigation of MDA production and enhanced *in vitro* antioxidant capacity (Li et al., 2011). Similarly, Jin et al. and Quan et al. utilized spectrophotometric analysis to demonstrate that BRPs dose-dependently inhibit lipid peroxidation in erythrocytes and erythrocyte membranes. These effects are attributed to the interruption of lipid peroxidation chain reactions and the preservation of cell membrane integrity, thereby conferring antioxidant and erythrocyte-protective effects (Jin Y. H. et al., 2011; Quan et al., 2011). Utilizing a rat model of senescence triggered by D-galactose, Piao et al. observed that crude ethanol extract of BR significantly bolstered cerebral SOD activity while simultaneously curbing MDA levels and monoamine oxidase (MAO) activity, and it improved hippocampal neuronal morphology. Such findings indicate that these polysaccharides enhance the endogenous antioxidant repertoire and alleviate oxidative stress-induced damage (Piao et al., 2003; Piao et al., 2004). To conclude, BRPs effectively alleviate oxidative stress-induced damage by terminating lipid peroxidation chains and amplifying antioxidant enzyme activity, thereby showing redox-related protective potential under experimental conditions. Figure 8 shows the antioxidant role of BRPs.

Overall, current research assesses the redox-related effects of BRPs based on indicators such as lipid peroxidation levels, SOD, MDA, NO, and MAO, alongside red blood cells, erythrocyte membranes, and D-galactose-induced aging animal models. Available data indicate that BRPs may alleviate lipid peroxidation and enhance endogenous antioxidant defenses under experimental settings. It should be noted that relevant evidence is largely derived from biochemical or *in vitro* tests and a small number of animal trials. Most existing work only records alterations in the above biomarkers, while few studies have verified the direct molecular targets and detailed regulatory mechanisms of BRPs. Subsequent research ought to adopt more experimental schemes based on cellular damage models, animal oxidative stress models, and disease-related *in vivo* platforms. On the premise of utilizing standardized polysaccharide fractions alongside complete structural characteristic data, further explorations are needed to elaborate the actual pharmacological significance of BRPs in exerting redox protective effects.

### 9.5 Hepatoprotective effects

Liver diseases are a major global health concern. Natural products derived from plants have garnered significant attention owing to their promising hepatoprotective effects with relatively low hepatotoxicity (Quan, 2015). Jin et al. established an H_2_O_2_-induced oxidative injury model in HL-7702 normal human hepatocytes and found that BRPs exert a regulatory influence on the MAPK and NF-κB. Specifically, they inhibit the phosphorylation of JNK and ERK, downregulate pro-apoptotic markers such as cleaved caspase-3, and diminish the Bax/Bcl-2 ratio, thereby attenuating oxidative stress-induced apoptosis and maintaining hepatocellular structure and function (Jin et al., 2021). BRPs also exhibit protective effects against H_2_O_2_-mediated oxidative damage in hepatocytes *in vitro*, as evidenced by increased cell viability; lowered alanine aminotransferase (ALT), aspartate aminotransferase (AST), and LDH activities; elevated SOD activity and glutathione (GSH) content; and reduced MDA levels (Yin et al., 2017). In a CCl_4_-induced acute liver injury model, Quan et al. found that polysaccharides from BR reduced serum ALT, AST, and alkaline phosphatase (ALP) levels. They also enhanced hepatic antioxidant capacity by increasing GSH content and SOD activity, while decreasing lipid peroxidation products such as MDA. In addition, the polysaccharides alleviated inflammatory responses, as evidenced by reduced serum TNF-α levels, decreased hepatic NO production, and downregulation of inducible nitric oxide synthase (iNOS) and cyclooxygenase-2 (COX-2) expression (Quan et al., 2013; 2014). Collectively, these findings indicate that BRPs exert hepatoprotective effects through anti-apoptotic, anti-inflammatory, and antioxidant mechanisms.

In contrast to basic chemical antioxidant screening assays, cellular and *in vivo* liver injury animal models can generate experimental outcomes that are more closely linked to practical pharmacological mechanisms. Cumulative research observations indicate that BRPs exert liver-protective effects mainly by modulating antioxidant defense systems, inflammatory signaling cascades, and apoptotic biological processes. Yet, most published works merely detected routine biochemical indicators such as ALT, AST, SOD, MDA, and TNF-α. In addition, relevant studies still lack standardized preparation protocols for polysaccharide extracts and systematic exploration of dose–response trends. Subsequent experimental schemes need to place more emphasis on histopathological scoring evaluation, rational positive control design, and functional validation of structurally characterized polysaccharide fractions, along with long-term safety surveillance assessments.

### 9.6 Other pharmacological effects

Beyond the biological activities discussed earlier, BRPs possess notable anti-aging and cardioprotective properties. Aging is associated with increased inflammatory responses and oxidative imbalance, and it often precipitates a spectrum of geriatric pathologies, ranging from malignancy and osteoporosis to cardiovascular and neurodegenerative ailments (Piao et al., 2003; Yang et al., 2024). BRPs exhibit anti-aging effects by enhancing antioxidant defenses and reducing oxidative damage. Mechanistically, they increase SOD activity in brain tissue, decrease MDA levels, and inhibit MAO activity, indicating their potential to alleviate oxidative stress and age-related functional decline. Furthermore, Chen et al. suggest that BRPs shield cardiomyocytes against injury. Mechanistically, BRPs exert cardioprotective effects by downregulating miR-302a-3p and upregulating its target gene, LRP8, thereby inhibiting hypoxia-induced oxidative stress and apoptosis in cardiomyocytes (Chen S. K. et al., 2023).

## 10 Applications

**FIGURE 9:**
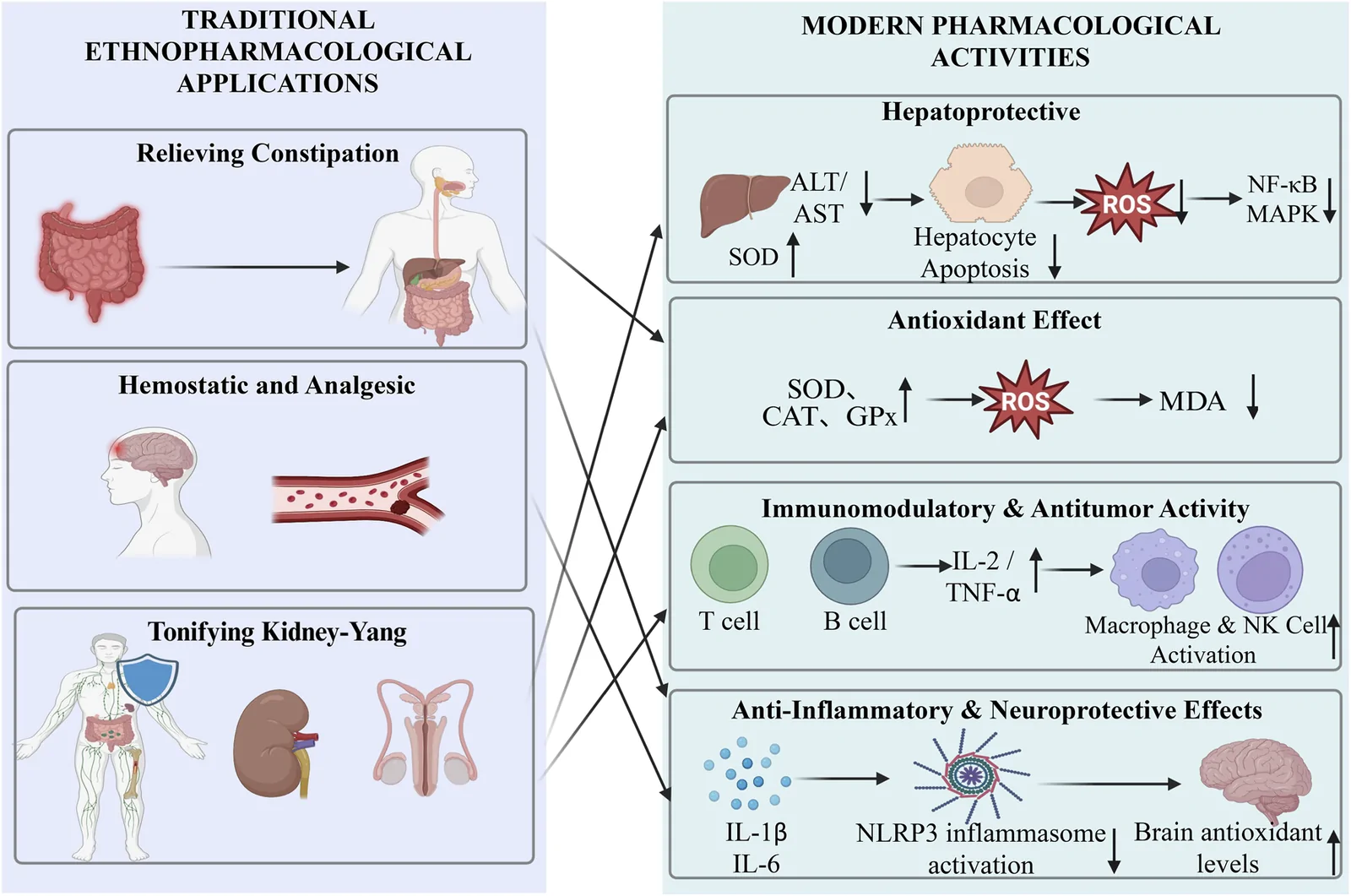
Relationship between the traditional medical applications and modern pharmacological activities of BRPs.

On the basis of both traditional usage and modern pharmacological studies, BR and its associated polysaccharides have emerged as focal points of scientific inquiry (Figure 9). Their multifaceted potential spans a broad spectrum of utility, particularly in the formulation of functional dietary products, nutraceutical agents, and novel drug discovery. With a long-standing use in traditional medicine, BR has been commonly used to increase physical strength and relieve symptoms related to aging and fatigue. In modern applications, it is frequently incorporated into functional foods and nutritional supplements, often in combination with other traditional Chinese medicinal herbs including Polygonatum plants, *Dioscorea opposita*, and *Lycium barbarum*. These combinations are generally used to support the body’s immune function and maintain the overall physiological balance (Chen Y. T. et al., 2023). Furthermore, products based on BR, such as Changbai Fuerchun capsules and Lubian Huichun capsules (Figures 1e,f), have been developed as dietary supplements with health-promoting properties (Long et al., 2019). These applications reflect the transition from traditional herbal use to standardized functional products. In addition to traditional and pharmaceutical uses, BRPs have been explored in the field of sports nutrition. Preliminary studies indicate that supplementation with BR extracts may improve exercise performance and reduce fatigue, with an acceptable safety profile (Jiang, 2016).

Although BRPs have shown good results in functional food development, nutritional supplements, and auxiliary natural medicinal products, large-scale industrial production of these polysaccharides has not been realized yet. First, a stable supply of BR raw materials cannot be guaranteed due to the limited geographical origins for growth and harvest, parasitic host varieties, and an annual harvesting cycle. At the same time, traditional hot water and thermal reflux extraction methods are slow in terms of processing time and are energy-intensive; furthermore, consistent extraction yields and uniform structural characteristics among different production batches have not been achieved. Third, BRPs are complex natural macromolecules whose molecular weight distribution, monosaccharide composition, uronic acid content, and branching degree are all affected by the preparation methods; thus, a single quality assessment standard cannot be established. Fourth, systematic studies have not been conducted on how BRPs maintain structural integrity during industrial processing, long-term storage, and gastrointestinal digestion; thus, their application in food matrices and pharmaceutical preparations is limited.

A set of feasible countermeasures has been proposed to address the above constraints for future research directions. The criteria for raw material origin control needs to be standardized, harvesting operations should be unified, and pre-treatment workflows should be regularized first. Second, to improve the extraction efficiency and reduce energy consumption, ultrasonic-enzymatic extraction, low-temperature short-time extraction, membrane extraction, and ultrafiltration fractionation technologies can be used. Third, an all-encompassing multi-index quality control system should be established to cover test indicators such as molecular weight distribution, monosaccharide ratios, characteristic glycosidic bonds, and chromatographic fingerprint spectra. Fourth, based on a full-scale structural elucidation and comprehensive biosafety assessments, mild chemical derivatization may be utilized to enhance the solubility, stability in storage, or biological activity of BRPs, such as carboxymethylation, sulfation, selenylation, and acetylation. Chemically modified derivatives are not structurally identical to the original BRPs, and for their industrial use, combined evaluations of biosafety, standardized quality monitoring, and relevant regulations are required. Fifth, encapsulation strategies such as microcapsule embedding, composite hydrogel systems, nanocarrier delivery, and food matrix loading can be utilized to enhance the structural stability of BRPs during production and storage. All of the above technical routes can promote the conversion of laboratory basic research into controllable and standardized industrial utilization of BRPs.

## 11 Conclusions and future perspectives

From an overall perspective, BRPs are natural polysaccharide substances that appear to have substantial room for subsequent development. Previous scholarly efforts have explored these compounds across multiple dimensions, including extraction and purification protocols, structural characterization, diverse documented biological activities, safety evaluation, and potential application scenarios. On the basis of systematically sorting out available published findings, the present review carries out comprehensive collation and analytical discussion surrounding BRPs following a core logical framework: how preparation techniques reshape structural properties, whether structural variations alter biological functions, and what research bottlenecks hinder translational exploitation. Distinct extraction and purification strategies are capable of changing multiple structural attributes of BRPs, such as molecular weight distribution, monosaccharide composition, uronic acid content, glycosidic linkage configurations, branching degrees, and high-order spatial conformations. Such structural discrepancies will further exert influences on a wide range of reported experimental bioactivities, including immunomodulation, inflammatory regulation, redox protective effects, liver protection, and proliferative suppression against tumor cells. Through the above systematic summarization, this work intends to deliver clearer references for standardized preparation schemes, in-depth mechanistic exploration, and follow-up translational development of BRPs.

Despite the above progress, many problems still exist in the current research on BRPs. First, the fine structural characterization of BRPs remains incomplete. Most of the research has focused on the large-scale structural characteristics, and specific information about small structures, such as exact linkers and their three-dimensional shape, is still lacking. Second, the structure–activity relationships have not yet been systematically identified. Polysaccharides are diverse in structure; therefore, it is difficult to know which structural traits correspond to which functions in life. Third, the specific molecular targets and receptor-binding sites of BRPs are still unknown, although some evidence has linked them to pathways such as MAPK, TLR4, and NF-κB. Most of the existing research is either *in vitro* or conducted on animals, and there is a shortage of high-quality clinical data to confirm its effectiveness and safety in people. Therefore, their clinical translational value has not yet been clearly established. Standardization of the extraction, purification, and quality control process for BRPs is also a problem at present, and it needs to be standardized to ensure that studies and products are reproducible and consistent.

Current research on BRPs largely relies on basic descriptive observations to characterize their bioactive properties; yet, upcoming studies can advance beyond such preliminary analyses. Future work would benefit from more systematic research schemes that probe the intrinsic mechanisms of BRP bioactivity, while advancing standardized quality regulation and pushing forward the translational application of existing laboratory outcomes. Establishing unified operational standards for BRP isolation, purification, and quality assessment is of great necessity for unifying scattered research systems. Researchers are advised to fully document the origin of raw materials, specific extraction parameters, and detailed purification workflows. Standardized experimental recording helps yield BRP samples with well-defined chemical structures and steady experimental reproducibility, which underpins subsequent in-depth exploration. Apart from cell and animal experiments, tools such as receptor inhibition, gene silencing, and pathway inhibitors are required to identify BRPs’ direct molecular targets, receptor-dependent regulatory pathways, and downstream signaling cascades that mediate biological functions. Finally, comprehensive evaluation of dose–effect curves, long-term safety, uniform quality standards, and clinical transformation prospects is essential. Research priority should be given to BRP components with definite structures, stable activity, and high safety, which can be further developed into functional food ingredients, nutritional supplements, and botanical auxiliary medicines.

## Glossary

ALP: Alkaline phosphatase
ALT: Alanine aminotransferase
Ara: Arabinose
AST: Aspartate aminotransferase
BJⅠ: BR polysaccharide component BJⅠ
BJⅡ: BR polysaccharide component BJⅡ
BR: Boschniakia rossica
BRPs: Boschniakia rossica Polysaccharides
BRT: BR polysaccharide component BRT
BWⅠ: BR polysaccharide component BWⅠ
BWⅡ: BR polysaccharide component BWⅡ
CCl₄: Carbon tetrachloride
CD: Cistanche deserticola
COX-2: Cyclooxygenase-2
DEAE: Diethylaminoethyl
DPPH: 1,1-Diphenyl-2-picrylhydrazyl
EAE: Enzyme-assisted extraction
EMT: Epithelial-mesenchymal transition
ERK: Extracellular regulated protein kinases
FT-IR: Fourier transform infrared spectroscopy
Fuc: Fucose
Gal: Galactose
GalA: Galacturonic acid
GC: Gas chromatography
Glc: Glucose
GlcA: Glucuronic acid
GSDMD-N: N-terminal fragment of gasdermin D
GSH: Glutathione
H₂O₂: Hydrogen peroxide
Hep-2: Human laryngeal carcinoma cell line
HepG2: Human hepatocellular carcinoma cell line
HIF-1α: Hypoxia-inducible factor-1α
HL-7702: Human normal hepatocyte cell line
HPLC: High-performance liquid chromatography
HPSEC: High-performance size-exclusion chromatography
IFN-γ: Interferon-γ
IL-1β: Interleukin-1β
IL-2: Interleukin-2
IL-6: Interleukin-6
iNOS: Inducible nitric oxide synthase
JNK: c-Jun N-terminal kinase
LDH: Lactate dehydrogenase
LPS: Lipopolysaccharide
Man: Mannose
MAO: Monoamine oxidase
MAPK: Mitogen-activated protein kinase
MDA: Malondialdehyde
Mw: Molecular weight
MyD88: Myeloid differentiation primary response 88
NF-κB: Nuclear factor kappa-B
NK: Natural killer
NLRP3: NOD-like receptor thermal protein domain associated protein 3
NO: Nitric oxide
5-FU: 5-Fluorouracil
PD-1: Programmed cell death protein 1
PD-L1: Programmed cell death ligand 1
PRISMA: Preferred Reporting Items for Systematic Reviews and Meta-Analyses
ROS: Reactive oxygen species
RSM: Response Surface Methodology
SOD: Superoxide dismutase
TBA: Thiobarbituric acid
TLR: Toll-like receptor
TNF-α: Tumor necrosis factor-α
UAE: Ultrasound-assisted extraction
UAEE: Ultrasound-assisted enzymatic extraction
Xyl: Xylose
